# Epidemic modeling with heterogeneity and social diffusion

**DOI:** 10.1007/s00285-022-01861-w

**Published:** 2023-03-25

**Authors:** Henri Berestycki, Benoît Desjardins, Joshua S. Weitz, Jean-Marc Oury

**Affiliations:** 1 École des hautes études en sciences sociales and CNRS, CAMS, Paris, France; 2 Institute for Advanced Study, Hong Kong University of Science and Technology, Sai Kung, Hong Kong; 3 ENS Paris-Saclay, CNRS, Centre Borelli, Université Paris-Saclay, 91190 Gif-sur-Yvette, France; 4 Geobiomics, 75 Av. des Champs Elysées, 75008 Paris, France; 5 School of Biological Sciences, Georgia Institute of Technology, Atlanta, GA USA; 6 School of Physics, Georgia Institute of Technology, Atlanta, GA USA; 7 Institut de Biologie, École Normale Supérieure, Paris, France

**Keywords:** Epidemiology, COVID-19, *SIR* model, Fokker–Planck equation, Reaction–diffusion system, Non-linear differential system, Heterogeneity, Social diffusion, 92D30, 35K57, 35Q92, 35Q84, 60J60, 92C60

## Abstract

We propose and analyze a family of epidemiological models that extend the classic Susceptible-Infectious-Recovered/Removed (SIR)-like framework to account for dynamic heterogeneity in infection risk. The family of models takes the form of a system of reaction–diffusion equations given populations structured by heterogeneous susceptibility to infection. These models describe the evolution of population-level macroscopic quantities *S*, *I*, *R* as in the classical case coupled with a microscopic variable *f*, giving the distribution of individual behavior in terms of exposure to contagion in the population of susceptibles. The reaction terms represent the impact of sculpting the distribution of susceptibles by the infection process. The diffusion and drift terms that appear in a Fokker–Planck type equation represent the impact of behavior change both during and in the absence of an epidemic. We first study the mathematical foundations of this system of reaction–diffusion equations and prove a number of its properties. In particular, we show that the system will converge back to the unique equilibrium distribution after an epidemic outbreak. We then derive a simpler system by seeking self-similar solutions to the reaction–diffusion equations in the case of Gaussian profiles. Notably, these self-similar solutions lead to a system of ordinary differential equations including classic SIR-like compartments and a new feature: the average risk level in the remaining susceptible population. We show that the simplified system exhibits a rich dynamical structure during epidemics, including plateaus, shoulders, rebounds and oscillations. Finally, we offer perspectives and caveats on ways that this family of models can help interpret the non-canonical dynamics of emerging infectious diseases, including COVID-19.

## Introduction

### Objectives of this paper

In this paper we propose, derive, and study a family of epidemiological models that extend the classic Kermack-McKendrick models of epidemics by structuring populations according to individual-level infection risk. The infection process modulates both the number of individuals in different compartments (e.g., susceptibles, infected, or recovered) as well as the infection risk amongst individuals who remain susceptible. The family of epidemiological models also includes a social diffusion mechanism by which infection risk changes both during and in the absence of epidemics. In doing so, the present work extends a previous model framework (Berestycki et al. 2021), proposed by a subset of the present author group. This prior article introduced a reaction–diffusion system to model epidemics in populations structured by heterogeneous infection risk, yielding non-canonical dynamics including plateaus, shoulders, and rebounds. However, this prior article only considered a particular framework with constant diffusion and no drift and did not provide a derivation of the model. Furthermore, the prior article did not include restorative mechanisms by which infection risk could stabilize during and after epidemics—as we will show, this feature provides the basis for both generalizations and useful simplifications in real-world contexts.

The scope of the study is as follows. First, in Sect. 2, we derive a general family of epidemiological models including heterogeneity in dynamic infection risk, extending the model proposed in Berestycki et al. (2021). Our initial analysis focuses on the evolution of the distribution of the risk parameter in the susceptible population. For this purpose, we represent the effect of the epidemic as a transport phenomenon that drives down the mean risk parameter through a process we term “sculpting”, consistent with similar proposals in Rose et al. (2021). The distribution is also sculpted by a mechanism of social diffusion that we represent as random variation with a reversion drift that are associated to a drift-diffusion stochastic process.

This *SfIR*–model describes the evolution of macroscopic (or bulk) quantities: *S*(*t*), *I*(*t*) and *R*(*t*), as in the classical framework, coupled with a microscopic description of the probability distribution function of risk behavior in the susceptible population that we denote *f*(*t*, *a*), where *a* is a risk factor variable. We are thus led to a system coupling *SIR* dynamics with a Fokker–Planck equation for the distribution *f* .

The *SfIR*–model admits a unique equilibrium distribution of risk in the absence of an epidemic. Our analysis begins by showing in Sect. 3 that the risk distribution will converge from an arbitrary initial distribution to this unique equilibrium distribution after the passage of an epidemic (Sect. 3). This section focuses on mathematical features of convergence, both in a general case and in the case of Gaussian profiles. The key take-away from an epidemiological perspective is that there is a unique equilibrium for the distribution *f* associated with fixed points in which the disease is not circulating, i.e., $I^{*}=0$. This unique equilibrium distribution is structured by parameters of social diffusion—balancing dynamic variations in infection risk with a tendency for individuals to reduce their infection risk subject to a minimum constraint. Precisely because the PDE model involves several parameters it is desirable to identify a reduced number of governing parameters to aid analysis and to apply model insights to real-world outbreaks. Hence, we look for parsimonious versions of the family of models that still retain key dynamical features of the full PDE model.

In Sect. 4, we derive such a parsimonious ODE system by assuming a Gaussian initial distribution of infection risk in the population. The number of free parameters in the ODE system is then considerably reduced with respect to the PDE model. This model reduction is achieved by looking for self-similar solutions of the system. The resulting ODE system includes the fraction of individuals in different disease compartments as in classical models (e.g., susceptibles, infectious, and recovered/removed), while augmenting these models with a state variable that represents the mean infection risk of individuals who remain susceptible (similar to an approach in the absence of social diffusion shown in Rose et al. 2021). In this way, the ODE system includes a statistical property of the complete distribution modeled in the PDE system. We show in Sect. 4.4 that this ODE system gives rise to a rich variety of dynamical behaviors. Remarkably, while much simpler, the ODE system exhibits hallmark qualitative features of dynamical complexity as found in the full PDE model—including plateaus, shoulders, and oscillations. We also prove some key mathematical properties of this reduced system.

In Sect. 6 we further derive higher order models to account for more general initial distributions beyond the Gaussian lol. This is achieved by using a spectral method with the eigenfunctions of the harmonic oscillator for a better approximation of the PDE system.

As we show, the *SfIR* model generates non-canonical features of epidemic dynamics as a direct result of incorporating changes in susceptibility that take place on the time scale of disease spread. In future work we plan to study extensions of the model developed here to include the dynamics of multiple variants, spatial propagation, feedback between awareness and behavior change, and to improve model-data integration through connections with surveillance data including wastewater-based epidemiology.

### Background

*SIR* and related epidemic models categorize individuals in a population according to their disease status, e.g., susceptible, infectious, and recovered/removed. There is a long history of expanding such models to incorporate population-level heterogeneity in the risk of infection, transmission, and/or outcome, e.g., by further differentiating individuals based on age, job type, race, gender, and/or socioeconomic status, etc. (Auchincloss and Diez Roux 2008; Stroud et al. 2007; Ibuka 2016; Leung 2017; Zhang 2020; Brauer 2005; Prem et al. 2017). Extensions to *SIR* models that include such heterogeneity assume that relevant epidemic parameters and interactions vary across categories (e.g., between ages as in Prem et al. 2021). The SARS-CoV-2 pandemic has accelerated a diversification of modeling approaches to examine the basis for and consequences of heterogeneity for the emergence of an outbreak, the speed of population-level spread and the size and severity of the outbreak at the population-scale. A key motivating factor was the early observation of plateaus and shoulders in SARS-CoV-2 case data—shapes that are seemingly incompatible with classic predictions of *SIR*-like models in which qualitative changes in dynamics are driven by susceptible depletion (Weitz et al. 2020a). As a result, multiple categories of models have been proposed to reconcile apparent slowdowns in spread, plateaus, shoulders, and oscillations in epidemic dynamics. In doing so, we distinguish three main classes of approaches: (i) bottom-up structured epidemic models; (ii) top-down behavioral models; (iii) mesoscopic models.

Structured epidemic models differentiate individual characteristics, including interactions and behavior, allowing for the inclusion of impacts on transmission from different types of interventions (e.g., school closing, case isolation, physical distancing) (Ferguson et al. 2020; Di Domenico et al. 2020). In the absence of interventions, structured epidemic models are expected to exhibit a single epidemic peak followed by a susceptible decline and elimination, whereas interventions can lead to delays, oscillations, rebounds, and/or asymmetric peaks. Even in the absence of interventions, classic outcomes predicted from *SIR* models can change when populations are structured. A key early example along these lines extended *SIR*-like models to account for the impacts of age- and activity-structure on the size of the ongoing SARS-CoV-2 outbreak (Britton et al. 2020). Numerical simulations revealed that herd immunity thresholds and final sizes of simulated outbreaks were smaller than that expected from classical theory when accounting for age-dependent mixing and activity differences. Other papers used more detailed features of social contacts such as social distance, indoor/outdoor environment, and the cumulative duration of contacts (Béraud et al. 2015). Likewise, a series of previous works described population heterogeneity in terms a finite number of transmission rate coefficients in the contact matrix in a *SIR* or *SEIR* modeling framework. (See for instance Arino et al. 2005; Dolbeault and Turinici 2020, 2021; Magal et al. 2018.) In all of these cases, non-homogeneous interactions in a structured population leads, in effect, to changes in transmission rates relative to those in homogeneous populations.

In contrast, top-down behavioral models neglect individual differences; instead relevant epidemic parameters change over time as a result of time-dependent rates or explicit feedback between spread, awareness, and behavior change. The merits and limits of these contrasting approaches to epidemic modeling are listed in Sukumar and Nutaro (2012). For example, in epidemic models with awareness-based feedback increasing fatalities leads to a decrease in interactions that correspondingly decreases transmission (Weitz et al. 2020a). In effect, the transmission rate $\beta$ is directly modulated by the number of cumulative (or daily) deaths. Depending on the degree of awareness, this model class can yield plateaus, “shoulder” like patterns and oscillations for the dynamics of infectious individuals—initial peaks are unrelated to susceptible depletion. Whether or not a population experiences, oscillations, a single peak, or sustained plateau in new infections may depend on the extent to which individuals become fatigued or substitute actions (e.g., mask-wearing or increasing ventilation) even as mobility increases. Similarly, other models have assumed that individuals face a behavioral trade-off described as a mathematical utility function (Arthur et al. 2021). In the absence of an epidemic, people try to improve their utility though social interactions—working, attending school, or socializing—so as to reach an ideal level of social contact. In the presence of an epidemic, interactions becomes risky. People then cut back their contacts to a level that balances the benefit of interactions with costs associated with catching the disease. The model is expressed as a *SIS* system where the transmission rate depends on the optimal contact rate. In this framework, there is a theoretical endemic equilibrium, which means that multiple fluctuation waves are possible for a long time around equilibrium values.

Finally, mesoscopic models represent an effort to bridge the gap between bottom-up agent-based modeling and top-down imposition of behavioral feedback in an otherwise homogeneous population. Mesoscopic models structure populations via an explicit representation of a distribution of traits that play a role in the infection process. Thus, these mesoscopic models are of an intermediate type in terms of complexity and dimensionality relative to the bottom-up and top-down modeling frameworks.

For example, in Rose et al. (2021), the authors incorporate population-level heterogeneity in infection susceptibility. They show that behavioral variation strongly influences the rate of infection, while the infection process simultaneously sculpts the susceptibility distribution. In doing so, they show how the sculpting process can drive distributions towards a characteristic stable shape (e.g., gamma-type susceptibility distributions). Precisely because individuals with greater susceptibility are infected earlier, the remaining population tends to be less susceptible than initially. As a result, the epidemic slows down, leading naturally to power-law behavior in the strength of infection, where the power-law like behavior is generic while the power-law exponent depends on the shape of the heterogeneous distribution. This work suggests that first-order epidemic models that are parameterized in the exponential-like phase may systematically over-estimate the final size and severity of the epidemic, similar to findings by Brauer (2011, 2019) and by Eksin et al. (2019). However, because individuals do not change their behavior, the model in Rose et al. (2021) does not generate oscillations or long-term plateaus. (See Tkachenko et al. 2021 for an effort to link dynamical heterogeneity to the finding of plateaus and multiple waves.)

There are multiple other examples—including Almeida et al. (2021) who introduced social diffusion together with behavior heterogeneity as a multidimensional risk variable *x* in a *SIR*-type framework. In this model, social diffusion appears as a linear diffusion of susceptible and infected populations (or only infected populations) in terms of *x*. Such models follow the kinetic modeling approach where the *SIR* (or *SEIR*) dynamics are coupled with Boltzmann or Fokker–Planck type equations and local equilibria being expressed as gamma-type distributions.

One may view the present work in the spirit of mesoscopic models. As such, we provide here a comprehensive and consistent derivation of a family of epidemiological models of reduced complexity encompassing both individual level risk heterogeneity and variability of individual behaviors (rather than variability in aggregate behaviors of the population as a whole). We term this a *SfIR* family of models. Such models describe the evolution of the distribution of population level quantities (*S*, *I*, and *R*) and the dynamics of a microscopic distribution *f* that captures variability in susceptibility to infection at the individual scale.

## The epidemiological model with heterogeneous and variable behaviors

### Stochastic framework

The model we introduced in Berestycki et al. (2021) involves a risk trait variable $a\in (0,1)$. More generally, here we will assume that *a* varies either in $A={\mathbb {R}}^+$ or in $A=(0,1)$. This parameter structures the population of susceptibles *S* so that $S=S(t,a)$. We can think of *a* as a lumped variable characterizing the relative vulnerability of individuals to infection. Low levels of *a* are associated with cautious behaviors while high values correspond to increasing risk of infection. Here, cautious behavior involves both the number of contacts and the probability of infection in a given contact. However, in our approach, we do not explicitly include the effect of cautious individuals reducing contacts with others, perceived as more at risk. This effect of *preferential mixing*, described in the work of Feng (2014), which also appears in the work of Weitz et al. (2020b), would lead to a different term for the force of infection.

The lumped variable *a* influences both the transmission rate factor profile $a \mapsto \beta (a)$ and the initial distribution of susceptible individuals: $a \mapsto S_0(a)= S(0,a)$. The effect of *a* is to yield high levels of transmission rates $\beta (a)$ for high values of *a* and small ones when *a* is small. Thus we always assume that $\beta (a)$ is increasing with *a*. Because behaviors fluctuate in time, it is natural to consider *a* as a random variable.

Our purpose in this section is to provide a detailed derivation of the generalized *SIR* system in the framework of a population structured by the variable *a*. The dynamics of the distribution of the variable *a* in the susceptible population results from two joint effects. First, by the very nature of the risk factor, individuals with high *a* become infected at a higher rate than those with a low level of *a*. Thus, the epidemics has a dynamic effect on the probability distribution of *a*. Second, owing to the stochastic nature of the parameter *a*, it is subject to its own stochastic evolution which we describe below by the stochastic differential equation (9). Thus, our generalized model describes the coupled evolution of behavior and epidemic dynamics.

### Dynamics of risk parameter *a* induced by heterogeneity: transport equation and Lagrangian viewpoint

We first derive the purely epidemic evolution of *a*. It can be considered as deterministic, in the framework of heterogeneous population with respect to disease transmission rate $a \mapsto \beta (a)$. In particular we aim at determining the corresponding transport equation for the sculpting of the distribution of susceptibles.

In an heterogeneous population, stratified by the risk parameter $a \in A$, (where *A* denotes either (0, 1) or ${\mathbb {R}}^+$), the purely epidemic depletion of *S*(*t*, *a*) is given by the first equation of the *SIR* model:

1 $$\begin{aligned} \frac{\partial S(t,a)}{\partial t} = - \frac{I(t)}{N} \beta (a) S(t,a). \end{aligned}$$

Here, we only consider the stratification of the population of susceptibles. As was already mentioned in Berestycki et al. (2021), one can also envision a situation in which the population of infected is structured by a parameter *b* and write $I=I(t,b)$. We leave this for further studies.

We introduce the probability density or distribution *f*(*t*, *a*) of the population of susceptibles as a function of the level *a* at time *t*. That is, we write:

$$\begin{aligned} \displaystyle f(t,a) := \frac{S(t,a)}{{\overline{S}}(t)}, \qquad \text {where} \qquad {\overline{S}}(t) := \int _A S(t,a) \, da. \end{aligned}$$

Thus, ${\overline{S}}(t)$ denotes the total susceptible population at time *t*.

From the heterogeneous *SIR* equation (1) above, we get

2 $$\begin{aligned} \frac{ d {\overline{S}}(t)}{dt} = - \frac{I(t) {\overline{S}}(t)}{N} \int _{A} \beta (a) f(t,a) \, da, \end{aligned}$$

and

3 $$\begin{aligned} \frac{\partial f(t,a)}{\partial t} = - \frac{I(t)}{N} f(t,a) \left( \beta (a) - \int _{A} \beta (b) f(t,b) \, db \right) . \end{aligned}$$

The meaning of (3) is clear. The effect of the epidemic is to deplete the fraction of population with high *a* and to increase the fraction of population *a* whose $\beta =\beta (a)$ is below the average $\beta$ in the population. Let *F* denote the cumulative distribution function, that is

$$\begin{aligned} F(t,a) = \int _0^a f(t,b) \, db. \end{aligned}$$

Integrating the right hand side of Eq. (3) with respect to *a* yields:

4 $$\begin{aligned} \frac{\partial f}{\partial t} = - \frac{\partial }{\partial a} \left( \mu _{ep} f \right) , \end{aligned}$$

where

5 $$\begin{aligned} \mu _{ep}(t,a) = \frac{I(t)}{N} \frac{\displaystyle \int _0^a \beta (b) f(t,b) \, db - F(t,a) \int _{A} \beta (b) f(t,b) \, db}{f(t,a)}\; , \end{aligned}$$

so that denoting ${\overline{\beta }}(t)$ the average transmission rate at time *t*, one has

6 $$\begin{aligned} \mu _{ep}(t,a) = \frac{I(t)}{N} \frac{\displaystyle \int _0^a \left( \beta (b) - {\overline{\beta }}(t) \right) f(t,b) \, db}{f(t,a)}\; . \end{aligned}$$

Equation (4) is a *transport equation*. It describes how the distribution of population *S*(*t*, *a*) reorganizes itself under the effect of the epidemics. Note that in the neighborhood of $a=0$, one has $\mu _{ep}(t,a) \sim a (\beta (0) - {\overline{\beta }}(t)) I(t)/N$.

Then, to represent the solutions of the transport Eq. (4), we use the method of characteristics. To this end, we introduce the associated Lagrangian flow $(t, \alpha ) \mapsto a(t,\alpha )$ that follows the dynamics

7 $$\begin{aligned} \frac{\partial a(t, \alpha )}{ \partial t} = \mu _{ep}\left( t,a (t, \alpha ) \right) , \quad \quad \hbox {with} \quad \quad a(0,\alpha ) = \alpha . \end{aligned}$$

As a matter of fact, one has

$$\begin{aligned} \frac{d }{dt} \left[ f(t,a(t,\alpha )) \partial _\alpha a(t,\alpha ) \right] = \partial _\alpha a(t,\alpha ) \left( \frac{\partial f}{\partial t} + \mu _{ep} \frac{\partial f}{\partial a} + f \frac{\partial \mu _{ep}}{\partial a}\right) (t,a(t,\alpha )) = 0. \end{aligned}$$

Hence, introducing the initial probability distribution $f_0$ and denoting $a^{-1}(t,\cdot )$ the inverse of $a(t,\cdot )$ for all given $t>0$, one gets

$$\begin{aligned} f(t,\alpha ) = \partial _\alpha a^{-1}(t,\alpha ) f_0(a^{-1}(t,\alpha )). \end{aligned}$$

This means that the probability measure of susceptibles ${\mathbb {P}}(t)$ (associated with the density $f(t,\cdot )$) at time *t* is the pushforward of the initial probability measure ${\mathbb {P}}_0$ by the flow $a(t,\cdot )$:

$$\begin{aligned} {\mathbb {P}}(t) = a(t,\cdot )_{\sharp } {\mathbb {P}}_0, \end{aligned}$$

That is, for all $G \subset A$, the measure of *G* at time *t* is deduced from the measure of the image of *G* by the inverse of the flow $a^{-1}(t, \cdot )$:

$$\begin{aligned} {\mathbb {P}}\left( t \right) \left( G\right) = {\mathbb {P}}_0 \left( a^{-1}\left( t,G\right) \right) . \end{aligned}$$

#### Remarks

Let us first observe that the right hand side $\mu _{ep}$ of (7) is always non-positive as soon as $\beta$ is a non-decreasing function of *a*. In fact, one has

$$\begin{aligned}{} & {} \int _0^a \beta (b) f(t,b) \, db - F(t,a) \int _{A} \beta (b) f(t,b) \, db \qquad \qquad \\{} & {} \qquad = \int _{A \setminus (0,a)} dc \int _0^a db \, f(t,b)f(t,c) \left( \beta (b) - \beta (c) \right) \le 0, \end{aligned}$$

where $A\setminus (0,a)$ denotes (*a*, 1) (resp. $(a,+\infty )$) when $A=(0,1)$ (resp. $A=(0,+\infty )$). This means that the risk *a* decreases along the Lagrangian characteristic curves governed by (7).

Another useful observation is that the right hand side $\mu _{ep}(t,a)$ of (7) vanishes for $a \in \partial A$, i.e. for $a=0$ and for $a=1$ (resp. $a \rightarrow +\infty$) in the case $A=(0,1)$ (resp. $A=(0,+\infty )$).

As in the *SIR* model, using the dynamics of *R* compartment ($dR/dt = \gamma I$), we deduce that the Lagrangian paths associated with (5) can be naturally expressed in terms of *R* instead of *t*:

8 $$\begin{aligned} \frac{d a}{d R} = \frac{\displaystyle \int _0^a \beta (b) f(R,b) \,db - F(R,a) \int _{A} \beta (b) f(R,b) \,db}{ \gamma N f(R,a)}. \end{aligned}$$

In the case of homogeneous transmission rate $\beta (a) \equiv \beta$, the epidemic drift vanishes $\mu _{ep}(t,a) \equiv 0$: epidemics do not create heterogeneity if there is none initially.

Let us finally give a computable example of non-zero epidemic drift. Starting from the exponential distribution $f_0(a) = \exp (-a)$ over $A=(0,+\infty )$, $\beta (a) = \beta _1 a$ for some positive constant $\beta _1$, one gets:

$$\begin{aligned} f(R,a)= & {} \frac{\displaystyle f_0(a) \exp \left( - \frac{\beta _1 a R}{\gamma } \right) }{ \int _0^{+\infty } \displaystyle f_0(b) \exp \left( - \frac{\beta _1 b R}{\gamma N} \right) db},\\ f(R,a)= & {} \left( 1+\frac{\beta _1 R}{\gamma N} \right) \exp \left( - a\left( 1+\frac{\beta _1 R}{\gamma N} \right) \right) , \end{aligned}$$

so that the dynamical system (8) becomes

$$\begin{aligned} \frac{da}{dR} = - \frac{\beta _1 a }{\displaystyle \gamma N \left( 1+\frac{\beta _1 R}{\gamma N} \right) }, \end{aligned}$$

i.e.

$$\begin{aligned} a(R) = \frac{a(0)}{ \displaystyle 1 + \frac{\beta _1 R}{\gamma N} } . \end{aligned}$$

This example illustrates the aforementioned monotonicity of $t \mapsto a(t)$: in this case, traits decrease with the same rate regardless of their initial value. Then, since

$$\begin{aligned} \mu _{ep}(t,a) = - \frac{\beta _1 \gamma I(t)}{ \gamma N+ \beta _1 R(t) } a, \end{aligned}$$

the transport equation satisfied by the distribution *f*(*t*, *a*) rewrites as

$$\begin{aligned} \frac{\partial f(t,a) }{\partial t} = \frac{\partial }{\partial a} \left( \frac{\displaystyle \beta _1 \gamma I(t) a f(t,a)}{\displaystyle \gamma N+ \beta _1 R(t)}\right) . \end{aligned}$$

### Derivation of the model

In order to account for random fluctuations of the distribution of the variable *a*, we consider that it is governed by a stochastic process that we write as $a_t$. We will assume that, in the absence of epidemics, its distribution results from the following stochastic differential equation or Itô drift diffusion process:

9 $$\begin{aligned} da_t= \mu _b(t,a_t) dt + \sigma (t,a_t) dW_t, \end{aligned}$$

where $W_t$ is a standard Wiener process, $\sigma (t,a)>0$, and $\mu _b(t,a)$ represents background volatility and drift coefficients. This equation corresponds to a Lagrangian point of view and is the stochastic analogue of the deterministic part (7). We impose reflecting boundary conditions at the endpoints of the interval *A*, i.e. at $a \in \{ 0, 1 \}$ when $A=(0,1)$ or at $a=0$ when $A=(0, \infty )$.

Let us now consider a population stratified by the variable *a* subject to the combined effect of two effects: the stochastic differential equation (SDE) (9) on one hand and depletion caused by the epidemics driven by transport equation (4) (or equivalently) by the ordinary differential equation (ODE) (7) on the other hand. We assume that the superposition principle applies to these two effects. Then, under this joint effect, the evolution of *a* includes a drift or transport term with coefficient $\mu _{ep} + \mu _b$, whereas the stochastic part remains the same. Thus, the random process is given by the following SDE:

$$\begin{aligned} da_t = \left( \mu _{ep} + \mu _b \right) dt + \sigma dW_t. \end{aligned}$$

where:

- $\mu _b$ is an additional drift and the diffusion $\sigma \ge 0$ both possibly depending on *t* and *a*. The two variables $\mu _b$ and $\sigma$ need to comply with boundary conditions:
  $$\begin{aligned} \mu _b - \frac{1}{2} \frac{\partial \sigma ^2}{\partial a} =0 \quad \hbox {on} \quad {\mathbb {R}}^+ \times \partial A. \end{aligned}$$
- $W_t$ is a Wiener process with reflecting boundary conditions.

Then, the dynamics of the associated probability density *f*(*t*, *a*) is given by the following Fokker–Planck equation (or forward Kolmogorov equation):

$$\begin{aligned} \frac{\partial f}{\partial t} = - \frac{\partial }{\partial a} \left( (\mu _{ep} +\mu _b) f \right) + \frac{\partial ^2}{\partial a} \left( \frac{\sigma ^2}{2} f \right) \quad \hbox {in} \quad {\mathbb {R}}^+ \times A. \end{aligned}$$

For the sake of completeness, we sketch the proof. This will allow us to derive the boundary condition. Let $a \mapsto \phi (a)$ be a smooth function over *A*. Then Itô’s formula leads to:

10 $$\begin{aligned} d\phi (a_t) = \left( \left( \mu _{ep} + \mu _b \right) \frac{\partial \phi }{\partial a} + \frac{\sigma ^2}{2} \frac{\partial ^2 \phi }{\partial a^2} \right) dt + \frac{\partial \phi }{\partial a} \sigma \, dW_t. \end{aligned}$$

Then, taking the time derivative of the expectation of $\phi (t,a)$ yields on the one hand

$$\begin{aligned} \frac{d }{dt} {\mathbb {E}}[\phi (a_t)] = \frac{d }{dt} \int _A \phi (a) f(t,a) \, da = \int _A \phi \frac{\partial f}{\partial t} \, da, \end{aligned}$$

and on the other hand from (10)

$$\begin{aligned} \frac{d }{dt} {\mathbb {E}}[\phi (a_t)]= & {} \frac{ {\mathbb {E}}[d \phi (a_t)] }{dt} = {\mathbb {E}} \left[ \left( \mu _{ep} + \mu _b \right) \frac{\partial \phi }{\partial a} + \frac{\sigma ^2}{2} \frac{\partial ^2 \phi }{\partial a^2} \right] , \\= & {} \int _A f \left( \left( \mu _{ep} + \mu _b \right) \frac{\partial \phi }{\partial a} + \frac{\sigma ^2}{2} \frac{\partial ^2 \phi }{\partial a^2} \right) da. \end{aligned}$$

Hence, one has for all smooth enough $\phi$

$$\begin{aligned} \int _A \phi \frac{\partial f}{\partial t}da = \int _A f \left( \left( \mu _{ep} + \mu _b \right) \frac{\partial \phi }{\partial a} + \frac{\sigma ^2}{2} \frac{\partial ^2 \phi }{\partial a^2} \right) da, \end{aligned}$$

so that integrating by parts the right hand side of the above equation leads to

$$\begin{aligned} \int _A \phi \frac{\partial f}{\partial t} da= & {} - \int _A \phi \frac{\partial }{\partial a} \left( \left( \mu _{ep} + \mu _b \right) f\right) da - \int _{A} \frac{\partial \phi }{\partial a} \frac{\partial }{\partial a} \left( \frac{\sigma ^2}{2} f \right) da \\{} & {} \quad + \left[ \left( \mu _b + \mu _{ep} \right) \phi f + \frac{\sigma ^2}{2} f \frac{\partial \phi }{\partial a} \right] _0^{1 \hbox { or } + \infty }, \\= & {} \int _A \left( - \phi \frac{\partial }{\partial a} \left( \left( \mu _{ep} + \mu _b \right) f\right) + \phi \frac{\partial ^2}{\partial a} \left( \frac{\sigma ^2}{2} f \right) \right) da \\{} & {} \quad + \left[ \left( \mu _b + \mu _{ep} \right) \phi f + \frac{\sigma ^2}{2} f \frac{\partial \phi }{\partial a} - \phi f \frac{\partial }{\partial a} \left( \frac{\sigma ^2}{2}\right) - \phi \frac{\sigma ^2}{2} \frac{\partial f}{\partial a} \right] _0^{1 \hbox { or } + \infty }. \end{aligned}$$

Taking test functions $\phi$ compactly supported in *A*, one therefore gets the dynamics of the probability density $(t,a) \mapsto f(t,a)$ of risk parameter *a*

$$\begin{aligned} \frac{\partial f}{\partial t} = - \frac{\partial }{\partial a} \left( (\mu _{ep} +\mu _b) f \right) + \frac{\partial ^2}{\partial a} \left( \frac{\sigma ^2}{2} f \right) \quad \hbox {in} \quad {\mathbb {R}}^+ \times A. \end{aligned}$$

Given the above assumptions on $\mu$ and $\sigma$ on the boundary $\partial A$, the following boundary conditions lead to a consistent weak formulation

$$\begin{aligned} \frac{\partial f(t,a)}{\partial a} = 0 \quad \hbox {on} \quad {\mathbb {R}}^+ \times \partial A. \end{aligned}$$

In other words, one has

$$\begin{aligned}{} & {} \frac{\partial f(t,a)}{\partial t} \\{} & {} \quad = -\frac{I(t)}{N} \left( \beta (a) f(t,a) \! - \! f(t,a) \! \int _{A} \!\beta (b) f(t,b) db \right) - \frac{\partial }{\partial a} \left( \mu _b f \right) + \frac{\partial ^2}{\partial a} \left( \frac{\sigma ^2}{2}f \right) . \end{aligned}$$

Using (2), which is unchanged (the effect of random variations reshuffle *a* in a conservative way), we deduce that $S(t,a) = {\overline{S}}(t) f(t,a)$ satisfies

$$\begin{aligned} \frac{\partial S(t,a)}{\partial t}= & {} {\overline{S}}(t) \frac{\partial f(t,a)}{\partial t} - f(t,a) \frac{I(t) {\overline{S}}(t)}{N} \int _{A} \beta (a) f(t,a) \, da, \\= & {} - \frac{I(t) \beta (a) S(t,a) }{N} - \frac{\partial }{\partial a} \left( \mu _b(t,a) S(t,a) \right) + \frac{\partial ^2}{\partial a} \left( \frac{\sigma ^2(t,a)}{2} S(t,a) \right) . \end{aligned}$$

On the other hand, the dynamics of the total infected is not modified by random fluctuations of *a*:

$$\begin{aligned} \frac{ d I(t)}{dt} = I(t) \left( \frac{1}{N} \int _A \beta (a) S(t,a) \, da - \gamma \right) . \end{aligned}$$

We conclude by the expression of the full PDE system of epidemiology with heterogeneous and variable behaviors:

11 $$\begin{aligned} \left\{ \begin{aligned} \displaystyle \frac{\partial S}{\partial t}&= - \displaystyle \frac{ \beta I S}{N} - \frac{\partial ( \mu _b S ) }{\partial a} + \frac{1}{2} \frac{ \partial ^2 (\sigma ^2 S) }{ \partial a^2} \\ \displaystyle \frac{d I}{dt}&= \displaystyle I \left( \frac{1}{N} \int _{A} \beta S \, da - \gamma \right) \\ \displaystyle \frac{dR}{dt}&= \gamma I \end{aligned} \right. \end{aligned}$$

supplemented with boundary conditions

$$\begin{aligned} \frac{\partial S}{\partial a} = 0 \qquad \hbox {on} \quad {\mathbb {R}}^+ \times \partial A, \end{aligned}$$

where $\mu$ and $\sigma$ are given functions satisfying the boundary conditions

$$\begin{aligned} \mu _b-\frac{1}{2} \frac{\partial \sigma ^2}{\partial a} = 0 \quad \hbox {on} \quad {\mathbb {R}}^+ \times \partial A. \end{aligned}$$

We recover in particular the model we studied in our earlier work (Berestycki et al. 2021) from the case $\mu _b\equiv 0$ and $\sigma$ constant. Note that we mentioned there the more general form of the equation when $\sigma =\sigma (a)$ depends on the risk trait *a* but we did not explain precisely how to derive it.

### General *SfIR* epidemiologic model

To summarize, we rewrite the general system describing the dynamics of ${\overline{S}}$, *I* and *f* variables

12 $$\begin{aligned} \boxed {\left\{ \begin{aligned}&\frac{d {\overline{S}}}{dt} = - \frac{ {\overline{\beta }}\, {\overline{S}}I}{N}, \qquad \frac{d I}{dt} = \displaystyle I \left( \frac{{\overline{\beta }}\, {\overline{S}}}{N} - \gamma \right) , \qquad {\overline{\beta }}(t) = \int _{A} \beta (a) f(t,a)\, da, \\&\frac{\partial f(t,a)}{\partial t} = - \left( \beta (a) - {\overline{\beta }}(t) \right) \!f(t,a) \frac{I(t)}{N} \\&\qquad \qquad \qquad \qquad - \frac{\partial \left( \mu _b(t,a) f(t,a) \right) }{\partial a} + \frac{\partial ^2 }{\partial a^2} \left( \frac{\sigma ^2(t,a) f(t,a)}{2} \right) , \\&\frac{\partial f(t,a)}{\partial a} = 0 \ \ \hbox {for} \ \ (t,a) \in {\mathbb {R}}^+ \times \partial A, \quad \int _{A} f(t,a) \, da = 1 \ \ \hbox {for} \ \ t \in {\mathbb {R}}^+. \end{aligned} \right. } \end{aligned}$$

The model parameters are assumed to satisfy

13 $$\begin{aligned} \boxed { \left\{ \begin{aligned}&\gamma> 0, \\&{\overline{S}}(0)>0, \quad I(0)>0 \quad \hbox {such that} \quad {\overline{S}}(0) + I(0) \le N, \\&\beta (a) \ge 0, \quad \sigma (t,a) \ge 0 \quad \hbox {in} \quad {\mathbb {R}}^+ \times A. \\&\mu _b(t,a)-\frac{1}{2} \frac{\partial \sigma ^2(t,a)}{\partial a} = 0 \quad \hbox {over} \quad {\mathbb {R}}^+ \times \partial A. \\ \end{aligned} \right. } \end{aligned}$$

In spite of its simple appearance, the evolution equation for *f* in (12) is rather involved. In fact, this equation is non-linear, not only because of the term *fI* (as in the quadratic term in the *SIR* model), but also owing to the term ${\overline{\beta }}f$ since ${\overline{\beta }}$ is linear in *f*. Note also that because of this term, the equation is *non-local*.

## Convergence to equilibrium

There is a vast literature related to the convergence to equilibrium of solutions to the Fokker–Planck equations in the whole space ${\mathbb {R}}^d$, $d\ge 1$ starting with the seminal results of Bakry and Émery (1985). We refer the reader to Markowich and Villani (1999) for a survey as well as to the book of Bakry et al. (2014). These works about convergence to equilibrium chiefly rely on a so-called “hyper-contractivity” condition of D. Bakry and M. Emery. We describe below this condition on the coefficients. In vague terms, it guarantees the existence of a “spectral gap” for the Fokker–Planck operator, which is shown to yield exponential convergence to equilibrium distribution in $L^1$. A key quantity is the “relative entropy” ${{\mathcal {E}}}$ involving the probability distribution *f* and the equilibrium distribution that we also describe here. Then, one shows that ${{\mathcal {E}}}$ is bounded by its dissipation rate by using the logarithmic Sobolev inequality that we state below.

In Sect. 3.1, we establish the convergence to equilibrium of the distribution $f(t,\cdot )$ solution of the coupled *SfIR* system (12) under general assumptions on the coefficients and initial data. Among these, we assume a hyper-contractivity condition which is sufficient for a logarithmic Sobolev inequality to hold. Actually, we might have considered alternative hyper-contractivity conditions involving the Ricci curvature of the Riemannian structure underlying the Fokker–Planck operator as in Arnold et al. (2001) without epidemic coupling. We leave this for future work.

Then, in Sect. 3.2, we consider a more specific framework for the coefficients $\beta$, $\mu _b$ and *D* in which the equilibrium distribution is of Gaussian type. It allows for much simpler proofs, making the argument more transparent. Furthermore, it allows one to derive stronger results such as the convergence of the average transmission rate ${\overline{\beta }}(t)$. Besides these advantages, this Gaussian framework has interesting mathematical properties such as the existence of exact self-similar solutions. Searching for such self-similar solutions leads to low complexity system of ordinary differential equations (ODEs) instead of the PDE system (12). We develop this point of view in Sect. 4 at first order and then extend it to higher order approximations in Sect. 6.1 using spectral decomposition of the distribution *f*.

### The general case with time-independent coefficients

From now on, we assume that $A = {\mathbb {R}}^+$ and we consider the *SfIR* system (12) in the case when the drift and diffusion coefficients, $\mu _b \ \ \hbox {and} \ \ D= \sigma ^2/2$, do not depend on time.

We consider here solutions *S*, *f*, *I* and *R* of (12) with the following assumptions on the model coefficients

$$\begin{aligned} \left\{ \begin{aligned}&\gamma > 0, \\&\beta (a) \ge 0, \quad D(a) \ge 0 \quad \hbox {for} \quad a \in {\mathbb {R}}^+, \\&\mu _b(0)-D'(0) = 0. \end{aligned} \right. \end{aligned}$$

After the epidemic has peaked, one expects that the number of infected individuals *I*(*t*) will go to zero and that the probability density $f(t,\cdot )$ will converge to the equilibrium distribution. This distribution denoted $f_\infty$ is the unique stationary solution of System (12) in the absence of epidemic. It satisfies:

14 $$\begin{aligned} \left\{ \begin{aligned}&\frac{d}{da} \left( \mu _b(a) f_\infty (a) - \frac{d}{d a} \left( D(a) f_\infty (a) \right) \right) = 0 \ \hbox {in} \ \ {\mathbb {R}}^+, \\&f_\infty '(0) = 0 \ \ \hbox {and} \ \ \int _{\mathbb {R^+}} f_\infty (a) da=1. \end{aligned} \right. \end{aligned}$$

Let us introduce $a \mapsto \alpha (a)$ defined up to an additive constant by

$$\begin{aligned} \alpha '(a) = \frac{D'(a) - \mu _b(a)}{D(a)}. \end{aligned}$$

We assume that $a \mapsto \exp (- \alpha (a))$ is integrable over ${\mathbb {R}}^+$ and we set the constant in such a way that

$$\begin{aligned} \int _0^{+\infty } \exp ( - \alpha (a) ) \, da =1. \end{aligned}$$

Then, we deduce from (14) that

$$\begin{aligned} f_\infty (a) = \exp \left( - \alpha (a) \right) . \end{aligned}$$

Associated to this equilibrium probability density, we define the measure $\nu$ by $d\nu (a) = f_\infty (a) \, da$ and related $L^p$ functional spaces

$$\begin{aligned} L^p\left( {\mathbb {R}}^+;\nu \right) = \left\{ g \ ; \ \int _{{\mathbb {R}}^+} |g(a)|^p d\nu (a) < + \infty \right\} , \quad p \in [1,+\infty ). \end{aligned}$$

We now state some assumptions on the coefficients $\beta$, $\mu _b$ and *D* that we shall use later on to study the convergence to equilibrium. The first one requires that the average initial and asymptotic transmission rates are finite:

$$\begin{aligned}&(H1) \quad {\overline{\beta }}(0) \! = \! \int _{{\mathbb {R}}^+} \beta (a) f(0,a) \, da< + \infty \ \ \hbox {and} \ \ {\overline{\beta }}_\infty \! = \! \int _{{\mathbb {R}}^+} \beta (a) f_\infty (a) \, da < + \infty .&\\ \end{aligned}$$

Next, we assume that the diffusion coefficient is non-degenerate, that is:

$$\begin{aligned}&(H2) \quad \hbox {There exists} \ \ D_* >0 \ \ \hbox {such that} \ \ D(a) \ge D_* \ \ \hbox {for all} \ \ a \in {\mathbb {R}}^+.&\end{aligned}$$

In order to keep the average transmission rate bounded over time, we also require the following technical assumption: there exist positive constants $C_1$ and $C_2$ such that

$$\begin{aligned}&(H3) \quad \beta '(0) \ge 0, \ D(a) \beta ''(a) + \mu _b(a) \beta '(a) \le C_1 - C_2 \beta (a) \ \hbox {for all} \ a \in {\mathbb {R}}^+.&\end{aligned}$$

We will see that it is verified in the case we discuss in the next subsection.

Finally, the following Assumption (*H*4) is the Bakry–Emery hyper-contractivity condition (Bakry and Émery 1985; Bakry et al. 2014; Bakry 1994; Arnold et al. 2001; Courtade and Fathi 2020) which allows one to control the convergence rate to equilibrium of solutions of the Fokker–Planck equations in the absence of epidemic. It simply writes in terms of $a \mapsto \alpha (a)$ as:

$$\begin{aligned}&(H4) \quad \alpha ''(a) \ge \uplambda _1, \quad \text {for all} \quad a\in {\mathbb {R}}^+\quad \text {for some} \quad \uplambda _1>0.&\end{aligned}$$

Equivalently, we formulate (*H*4) in terms of the *D* and $\mu _b$ parameters as follows for all $a \in {\mathbb {R}}^+$:

$$\begin{aligned}&(H4) \quad \frac{D''(a)}{D(a)} -\frac{D'(a)^2}{D(a)^2} + \frac{\mu _b(a) D'(a)}{D(a)^2} - \frac{\mu _b'(a)}{D(a)} \ge \uplambda _1 >0.&\end{aligned}$$

A classical result stated in Bakry et al. (2014), Courtade and Fathi (2020) is the following Sobolev type estimate:

#### Theorem 1

(Bakry–Emery) Let $a \mapsto \alpha (a)$ be a twice differentiable function over ${\mathbb {R}}$ such that for some $\uplambda _1>0$, $\alpha ''(a) \ge \uplambda _1>0$ for all $a\in {\mathbb {R}}$, and $f_\infty = \exp (-\alpha )$ is a probability measure over ${\mathbb {R}}$. Then, defining the measure $\nu$ by $d \nu (a) = f_\infty (a) \, da$, for all $g \in L^2({\mathbb {R}}; \nu )$ such that $g'=dg/da \in L^2({\mathbb {R}}; \nu ),$ there holds:

15 $$\begin{aligned} \int _{{\mathbb {R}}} g^2 \log g^2 d \nu - \left( \int _{{\mathbb {R}}} g^2 d\nu \right) \log \left( \int _{{\mathbb {R}}} g^2 d\nu \right) \le \frac{2}{\uplambda _1} \int _{{\mathbb {R}}} | g'|^2 d\nu , \end{aligned}$$

As a consequence, for the probability density distributions *f* over ${\mathbb {R}}^+$, we obtain the following Logarithmic Sobolev inequality:

16 $$\begin{aligned} \int _{{\mathbb {R}}^+} f(a) \psi (a) \, da \le \frac{1}{2 \uplambda _1} \int _{{\mathbb {R}}^+} f(a) \left| \frac{d \psi (a)}{da} \right| ^2 da, \ \ \hbox {where} \ \ \psi = \log \frac{f}{f_\infty }. \end{aligned}$$

This inequality is easily derived from (15) by extending *f*/2 and $f_\infty /2$ as even functions on the whole real line, and considering $g= \sqrt{f / f_\infty }$. It holds true as long as $g'\in L^2({\mathbb {R}}^+; \nu )$.

The left hand side of (16) is called the relative entropy^1^. This inequality allows one to estimate this entropy by what turns out to be its dissipation rate through the dynamics of Fokker–Planck equations in the right hand side.

On the one hand, in the classical Fokker–Planck equation case, that is, in the absence of coupling with epidemic model, this Logarithmic Sobolev inequality leads to a convergence of $f(t,\cdot )$ to $f_\infty$ in $L^1({\mathbb {R}}^+)$ norm at speed $\exp (- \uplambda _1 t)$ (see Bakry and Émery 1985; Bakry 1994; Bakry et al. 2014). On the other hand, in the classical *SIR* model, in the absence of social diffusion, the quantities *S*(*t*), *I*(*t*) also converge exponentially to their limits as $t\rightarrow \infty$.

Here, the novelty lies in the *coupling* of the Fokker–Planck equation with the epidemiological system which is not covered by previous results. Still, we are able to prove the following result.

#### Theorem 2

Under Assumptions (H1)(H2)(H3)(H4), *I*(*t*) converges to 0, ${\overline{S}}(t)$ converges to ${\overline{S}}_\infty$ and $f(t,\cdot )$ converges to $f_\infty$ in $L^1({\mathbb {R}}^+)$ as time *t* goes to $+\infty$ for some ${\overline{S}}_\infty$ such that $0\le {\overline{S}}_\infty < {\overline{S}}(0)$. More precisely, assuming that $f_0 \log (f_0/f_\infty ) \in L^1({\mathbb {R}}^+)$, there exists $C_*>0$ such that for all $t>0$

17 $$\begin{aligned}{} & {} \left( \int _{{\mathbb {R}}^+} \left| f(t,a) - f_\infty (a)\right| da \right) ^2 \le C_* \int _0^t \frac{I(s)}{N} \exp (\eta (s-t)) \, ds \nonumber \\{} & {} \quad \qquad + 2 \exp (-\eta t) \int _{{\mathbb {R}}^+} f_0(a) \log \frac{ f_0(a)}{f_\infty (a)} da, \end{aligned}$$

where $$\eta = 2 D_* \uplambda _1$$.

Let us explain why (17) leads to the convergence in $L^1({\mathbb {R}}^+)$ of $f(t, \cdot )$ to $f_\infty$ as *t* goes to $+\infty$. As we shall see in the first step of the proof of Theorem 2, the convergence of ${\overline{S}}(t)$ (resp. *I*(*t*)) to ${\overline{S}}_\infty \in [0,{\overline{S}}(0))$ (resp. 0) follows from straightforward monotonicity arguments. Then, given $T>0$ and splitting the integral over (0, *t*) in the right hand side of (17) into (0, *T*) and (*T*, *t*) yields for $t>T$:

$$\begin{aligned} \int _0^t \frac{I(s)}{N} \exp (\eta (s-t)) \, ds \le \frac{1}{\eta } \left( \sup _{s \ge T} |I(s)| + \exp ( -\eta (t-T) ) \right) , \end{aligned}$$

which leads to the convergence in $L^1({\mathbb {R}}^+)$ of $f(t, \cdot )$.

We also observe that Estimate (17) means that the rate of convergence of the probability density *f* to equilibrium is determined by the smallest decay between that of *I*(*t*) and the exponential decay associated with the convergence rate to equilibrium solutions of the Fokker–Planck equations. As a matter of fact, using Fatou’s lemma, the convergence of $f(t,\cdot )$ to $f_\infty$ in $L^1({\mathbb {R}}^+)$ leads to:

$$\begin{aligned} {\overline{\beta }}_\infty = \int _{{\mathbb {R}}^+} \liminf _{t \rightarrow \infty } \beta (a) f(t,a) \, da \le \liminf _{t \rightarrow +\infty } {\overline{\beta }}(t). \end{aligned}$$

Therefore, we deduce from the convergence of ${\overline{S}}(t)$ to ${\overline{S}}_\infty$ that

$$\begin{aligned} {\overline{\beta }}_\infty {\overline{S}}_\infty - \gamma \le \liminf _{t \rightarrow +\infty } \left( {\overline{\beta }}(t) {\overline{S}}(t) - \gamma \right) . \end{aligned}$$

Recalling that $dI/dt = I ({\overline{\beta }}\, {\overline{S}}- \gamma )$, we deduce that if

$$\begin{aligned} {{\bar{\delta }}} = - \liminf _{t \rightarrow +\infty } \left( {\overline{\beta }}(t) {\overline{S}}(t) - \gamma \right) > 0, \end{aligned}$$

which requires ${\overline{\beta }}_\infty {\overline{S}}_\infty < \gamma$, then for all $\delta \in (0, {{\bar{\delta }}})$, there is some $C_\delta >0$ such that

$$\begin{aligned} I(t) \le C_\delta \exp ( - \delta t). \end{aligned}$$

Then, $L^1({\mathbb {R}}^+)$ convergence to equilibrium occurs at exponential speed

$$\begin{aligned} \int _{{\mathbb {R}}^+} \left| f(t,a) - f_\infty (a)\right| da \le C'_{*} \exp ( - \kappa t ), \end{aligned}$$

for some positive constant $C'_{*}$, where

$$\begin{aligned} \kappa = D_* \uplambda _1 \ \ \hbox {if} \ \ 2 D_* \uplambda _1 < {{\bar{\delta }}}, \quad \kappa \in (0, {{\bar{\delta }}} /2) \ \ \hbox {otherwise}. \end{aligned}$$

The case when ${\overline{\beta }}_\infty {\overline{S}}_\infty = \gamma$, however, is more intricate regarding the convergence speed of *I*(*t*) to 0. Still, Theorem 2 holds even if *I*(*t*) were to converge more slowly to zero than exponential decreasing functions.

We now turn to the proof of Theorem 2.

#### Proof

In order to prove the large time convergence of solutions $({\overline{S}}, \, I, \, f)$ of (12) to some equilibrium $({\overline{S}}_\infty , \, 0, \, f_\infty )$, we need to adapt the Lyapunov function introduced in the classical works in the absence of epidemic (Frank 2001). The so-called relative entropy

$$\begin{aligned} {{\mathcal {E}}}(t) = \int _{{\mathbb {R}}^+} f(t,a) \log \frac{f(t,a)}{f_\infty (a)} da \end{aligned}$$

plays the role of such a Lyapunov function. The first step consists in proving that $({\overline{S}}(t), I(t))$ converge. The second one is to prove that the average transmission rate $t \mapsto {\overline{\beta }}(t)$ is bounded. The third step is to derive relative entropy estimates in terms of its dissipation rate and the rate of infected individuals *I*(*t*)/*N*. Then logarithmic Sobolev inequality (16) and Csiszár-Kullback-Pinsker inequality will allow us to conclude.

#### Step 1: Convergence of $({\overline{S}}(t), I(t))$

From the evolution equations of ${\overline{S}}$ and *I*, we deduce that ${\overline{S}}(t) \ge 0$ and $I(t)\ge 0$ for all $t\ge 0$. If follows that ${\overline{S}}(t)$ is non-increasing with time *t* so that it converges to some ${\overline{S}}_\infty \in [0, {\overline{S}}(0))$. Introducing the recovered population

$$\begin{aligned} R(t) = N - {\overline{S}}(t) - I(t) = \gamma \int _0^t I(s) ds, \end{aligned}$$

which is bounded from above by *N* and non-decreasing, we see that *R*(*t*) converges to some $R_\infty$. We conclude that $I(t) = N - {\overline{S}}(t) - R(t)$ converges to some value ${{\bar{I}}}_\infty = N - {\overline{S}}_\infty - R_\infty$, which is zero (it would otherwise contradict the convergence of *R*(*t*)).

#### Step 2: Bounds on $t \mapsto {\overline{\beta }}(t)$

Recalling the epidemiological model, it is natural to expect that the average transmission rate ${\overline{\beta }}(t)$ should remain bounded as time goes to $+\infty$. Integrating the evolution equation of *f* multiplied by $\beta (a)$, we deduce that $t \mapsto {\overline{\beta }}(t)$ is governed by

$$\begin{aligned} \frac{d {\overline{\beta }}(t)}{dt} = - \frac{I(t)}{N} \hbox {Var } \beta (t) + \int _{{\mathbb {R}}^+} \beta '(a) \left( \mu _b(a) f(t,a) - \frac{\partial }{\partial a}\left( D(a) f(t,a) \right) \right) da, \end{aligned}$$

where we have used $\mu _b(0)= D'(0)$ and where

$$\begin{aligned} \hbox {Var } \beta (t) = \int _{{\mathbb {R}}^+} \left( \beta (a) - {\overline{\beta }}(t)\right) ^2 f(t,a) \, da. \end{aligned}$$

Then, one has

$$\begin{aligned}{} & {} \frac{d {\overline{\beta }}(t)}{dt} + \frac{I(t)}{N} \hbox {Var } \beta (t) + \beta '(0) D(0) f(t,0) \\{} & {} \qquad = \int _{{\mathbb {R}}^+}\left( \beta '(a) \mu _b(a) + \beta ''(a) D(a) \right) f(t,a) \, da. \end{aligned}$$

The above computations are formal. Their rigorous justification requires sufficient regularity and decay at infinity of solutions *f*. These aspects are proved in Appendix C.

Recalling that $f\ge 0$, $\hbox {Var} \, \beta (t) \ge 0$ and using the non-degeneracy assumption (*H*2) and Condition (*H*3), we deduce that

18 $$\begin{aligned} \frac{d{\overline{\beta }}(t)}{dt} \le C_1 - C_2 {\overline{\beta }}(t). \end{aligned}$$

Since ${\overline{\beta }}(0)<+\infty$ by (*H*1), we infer that $t \mapsto {\overline{\beta }}(t)$ is bounded by some positive constant *B* for all time $t\ge 0$.

#### Step 3: Relative entropy estimate

Next, we observe that the partial differential equation satisfied for *f* can be expressed in the following way involving $f_\infty$:

$$\begin{aligned} \frac{\partial f(t,a)}{\partial t} = \frac{\partial }{\partial a} \left( f(t,a) D(a) \frac{\partial \psi (t,a)}{\partial a} \right) - \left( \beta (a) - {\overline{\beta }}(t) \right) f(t,a) \frac{I(t)}{N}, \end{aligned}$$

where

$$\begin{aligned} \psi (t,a) = \log \frac{f(t,a)}{f_\infty (a)}. \end{aligned}$$

Using the fact that $\partial _a \psi (t,0) = 0$ and defining the non-negative function $x \mapsto \xi (x)$ for positive *x* as $\xi (x)=x \log x -x + 1$, integration by parts yields

3.1 $$\begin{aligned}&\frac{d}{dt} \int _{{\mathbb {R}}^+} f(t,a) \psi (t,a) \, da + \int _{{\mathbb {R}}^+} f(t,a) D(a) \left( \frac{\partial \psi (t,a)}{\partial a}\right) ^2 da \\&\qquad + \frac{I(t)}{N} \int _{{\mathbb {R}}^+} \beta (a) \xi \left( \frac{f(t,a)}{f_\infty (a)} \right) f_\infty (a)\, da \\&\quad = \frac{I(t)}{N} \left( {\overline{\beta }}_\infty - {\overline{\beta }}(t) \right) + {\overline{\beta }}(t) \frac{I(t)}{N} \int _{{\mathbb {R}}^+} f(t,a) \psi (t,a) \, da . \end{aligned}$$

Here we have used Assumption (*H*1). Again, we made formal computations requiring sufficient regularity and decay of *f* that are proved in Appendix C. We then infer from (3.1) that

$$\begin{aligned}{} & {} \frac{d}{dt} \int _{{\mathbb {R}}^+} f(t,a) \psi (t,a) \, da + \int _{{\mathbb {R}}^+} f(t,a) D(a) \left( \frac{\partial \psi (t,a)}{\partial a}\right) ^2 da \\{} & {} \quad \le \frac{I(t)}{N} \left( {\overline{\beta }}_\infty + {\overline{\beta }}(t) \int _{{\mathbb {R}}^+} f(t,a) \psi (t,a) \, da \right) . \end{aligned}$$

Therefore, we infer that

$$\begin{aligned}{} & {} \int _{{\mathbb {R}}^+} f(t,a) \psi (t,a) \, da \le \exp \left( \int _0^t {\overline{\beta }}(s) I(s) /N ds \right) \int _{{\mathbb {R}}^+} f(0,a) \psi (0,a) \, da \\{} & {} \quad + \int _0^t {\overline{\beta }}_\infty \frac{I(s)}{N} \exp \left( \int _s^t {\overline{\beta }}(\tau ) I(\tau ) /N d\tau \right) ds. \end{aligned}$$

Hence, for some positive constant $C_3$, using the fact that $R_\infty = \gamma \int _0^{+\infty } I(t) dt \le N$, there exists some constant $B>0$ such that for all time $t\ge 0$

$$\begin{aligned} \int _{{\mathbb {R}}^+} f(t,a) \psi (t,a) \, da \le \exp (B) \int _{{\mathbb {R}}^+} f(0,a) \psi (0,a) \, da + C_3 \exp (B). \end{aligned}$$

Going back to (3.1), we deduce that there exists a positive constant $C_*$ such that

19 $$\begin{aligned} \frac{d}{dt} \int _{{\mathbb {R}}^+} f(t,a) \psi (t,a) \, da + \int _{{\mathbb {R}}^+} f(t,a) D(a) \left( \frac{\partial \psi (t,a)}{\partial a}\right) ^2 da \le C_* \frac{I(t)}{N}. \end{aligned}$$

#### Step 4: Logarithmic Sobolev inequality

In the absence of epidemic, the following quantity is usually viewed as the relative entropy dissipation rate

$$\begin{aligned} \int _{{\mathbb {R}}^+} f(t,a) D(a) \left( \frac{\partial \psi (t,a)}{\partial a}\right) ^2 da. \end{aligned}$$

In order to estimate it, we make use of the Logarithmic Sobolev inequality stated in (16) and the lower bound on *D*(*a*) from Assumption (*H*2). Thus, we get

$$\begin{aligned} \int _{{\mathbb {R}}^+} f(t,a) \psi (t,a)\, da \le \frac{1}{2 D_* \uplambda _1} \int _{{\mathbb {R}}^+} f(t,a) D(a) \left( \frac{\partial \psi (t,a)}{\partial a}\right) ^2 da. \end{aligned}$$

From (19), we infer

$$\begin{aligned} \frac{d}{dt} \int _{{\mathbb {R}}^+} f(t,a) \psi (t,a) \, da + 2 D_* \uplambda _1 \int _{{\mathbb {R}}^+} f(t,a) \psi (t,a) \, da \le C_* \frac{I(t)}{N}, \end{aligned}$$

so that

$$\begin{aligned} \int _{{\mathbb {R}}^+} f(t,a) \psi (t,a) \, da \le \exp ( - \eta t) \int _{{\mathbb {R}}^+} f(0,a) \psi (0,a) \, da \end{aligned}$$

20 $$\begin{aligned} \qquad \qquad \qquad \qquad + C_* \int _0^t \exp (\eta (s-t)) \frac{I(s)}{N} ds, \end{aligned}$$

where $$\eta = 2 D_* \uplambda _1$$.

#### Step 5: Conclusion

In order to conclude, we rely on the Csiszár-Kullback-Pinsker inequality. It allows us to estimate the squared $L^1$ distance of the probability distribution $f(t,\cdot )$ to the equilibrium $f_\infty$ in terms of the relative entropy (Csiszár 1967; Kullback 1967; Pinsker 1964). ${\mathbb {R}}$.

##### Theorem 3

(Csiszár–Kullback–Pinsker) Let *f* and *g* be two non-negative real functions in $L^1({\mathbb {R}}^+)$ with $\Vert f\Vert _1=\Vert g\Vert _1 = 1$. Then, the following inequality holds:

21 $$\begin{aligned} \left\| f - g \right\| _1^2 \le 2 \int _{{\mathbb {R}}^+} f \log \frac{f}{g}. \end{aligned}$$

For the reader’s convenience and because most of the related references are stated in ${\mathbb {R}}$ and not in the half space ${\mathbb {R}}^+$, we recall a simple proof due to J.A. Canizo (https://canizo.org/page/28) in Appendix B. Using the above estimate (21), we conclude that

$$\begin{aligned} \left( \int _{{\mathbb {R}}^+} \left| f(t,a) - f_\infty (a)\right| da \right) ^2 \le 2 \int _{{\mathbb {R}}^+} f(t,a) \psi (t,a) \, da, \end{aligned}$$

so that (20) allows us to complete the proof of Theorem 2 up to a change of $C_*$ by a factor 2. $\square$

#### Additional results and comments

Given the convergence in $L^1({\mathbb {R}}^+)$ of the distribution $f(t,\cdot )$ to $f_\infty$, it is natural to expect that the average transmission rate ${\overline{\beta }}(t)$ will converge to ${\overline{\beta }}_\infty$. From the $L^1$ convergence property, Fatou’s Lemma yields :

$$\begin{aligned} {\overline{\beta }}_\infty \le \liminf _{t \rightarrow + \infty } {\overline{\beta }}(t). \end{aligned}$$

However, additional assumptions seem necessary to infer the convergence of ${\overline{\beta }}(t)$ to ${\overline{\beta }}_\infty$. For instance, assuming $C_1 \le C_2 {\overline{\beta }}_\infty$ yields this convergence where $C_1, C_2$ are the constants in condition (*H*3). Indeed, in that case, we infer from (18) that

$$\begin{aligned} {\overline{\beta }}(t) - {\overline{\beta }}_\infty \le \exp ( - C_2 t) ({\overline{\beta }}(0) - {\overline{\beta }}_\infty ), \end{aligned}$$

so that

$$\begin{aligned} \limsup _{t \rightarrow + \infty } {\overline{\beta }}(t) \le {\overline{\beta }}_\infty , \end{aligned}$$

and therefore $\lim _{t \rightarrow + \infty } {\overline{\beta }}(t) = {\overline{\beta }}_\infty$. Such an apparently *ad hoc* assumption nevertheless applies to some interesting situations described in Sect. 3.2.

#### Open problems

The analysis of long term behavior for the system *SfIR* we have provided here opens the way to many mathematical questions.

1. It is of interest to relax the (*H*2) condition and address the case of degenerate diffusion functions $a \mapsto D(a)$.
2. Condition (*H*4) to estimate the relative entropy in terms of its dissipation rate is not optimal. Following Arnold et al. (2001) in the absence of epidemiological coupling, one may replace (*H*4) by the following assumption (*H*5) leading to a more accurate convergence to equilibrium associated with the spectral gap of the uncoupled Fokker–Planck dynamical system: there exists $\uplambda _2 >0$ such that
  $$\begin{aligned}&(H5) \quad -\frac{1}{4} \frac{D'^2(a)}{D(a)} + \frac{D''(a)}{2} + \frac{\mu _b(a) D'(a)}{2 D(a)} - \mu _b'(a) \ge \uplambda _2 >0. \end{aligned}$$
  Note that in this assumption we do not require that *D* be non-degenerate. We expect to be able to derive the convergence Theorem 2 under this condition.
3. The approach extends naturally to the case when the trait *a* covers a *d* dimensional space, provided (*H*1)...(*H*4) assumptions incorporate suitable dependence on *d*.
4. The degenerate case where ${\overline{\beta }}_\infty {\overline{S}}_\infty = \gamma$ raises interesting open questions since large time exponential convergence of infected population *I*(*t*) to 0 does not seem to hold any longer. Describing the convergence of *I*(*t*) to zero is one of the open questions. Likewise, in this case, it would be of interest to identify the rate of convergence of *f* to $f_\infty$ in $L^1$.

### The Gaussian case

The previous subsection is rather technical and involves some intricate assumptions in order to prove the large time convergence of the probability distribution $f(t,\cdot )$ to equilibrium. In order to make the arguments of Theorem 2 more transparent, we provide a separate comprehensive proof in the Gaussian case. That is, we consider henceforth the following framework.

(i) The background drift term $\mu _b$ has linear dependence in *a*:
  $$\begin{aligned} \mu _b(t,a) = - \mu _0 a, \end{aligned}$$
  for some positive constant $\mu _0$. The coefficient $\sigma$ and hence the diffusion coefficient *D* are constant.
(ii) The transmission rate takes the form
  $$\begin{aligned} \beta (a) = \beta _0 + \beta _1 a^2. \end{aligned}$$
  where $\beta _0 \ge 0$ and $\beta _1>0$ are two constants.
(iii) The initial probability distribution $f_0$ has a finite second moment, that is:
  $$\begin{aligned} \int _{{\mathbb {R}}^+} a^2 f_0(a) \, da < + \infty . \end{aligned}$$

In the absence of epidemic, Assumption (i) means that the trait *a* satisfies the Ornstein-Uhlenbeck Stochastic Differential Equation (SDE)

22 $$\begin{aligned} da_t = - \mu _0 a_t dt + \sigma dW_t, \end{aligned}$$

with reflecting boundary condition at $a=0$. Denoting again $D=\sigma ^2/2$, the corresponding Fokker–Planck equation then writes:

$$\begin{aligned} \left\{ \begin{aligned}&\frac{\partial f(t,a)}{\partial t} = \mu _0 \frac{\partial \left( a f(t,a) \right) }{\partial a} + D \frac{\partial ^2 f(t,a)}{\partial a^2} \quad \hbox {for} \quad (t,a) \in {\mathbb {R}}^+ \times {\mathbb {R}}^+, \\&\partial _a f (t,0)=0 \quad \hbox {for} \quad t \in {\mathbb {R}}^+,\\&\int _{{\mathbb {R}}^+} f(t,a) \, da = 1 \quad \hbox {for} \quad t \in {\mathbb {R}}^+, \end{aligned} \right. \end{aligned}$$

with initial condition $f_0 \ge 0$ such that

$$\begin{aligned} \int _{{\mathbb {R}}^+} f_0(a) \, da = 1. \end{aligned}$$

In the presence of epidemic propagation, (22) is replaced by

$$\begin{aligned} da_t = \left( \mu _{ep}(t,a_t)- \mu _0 a_t\right) dt + \sigma dW_t. \end{aligned}$$

Then, using (6), the coupled model (12) in terms of the variables ${\overline{S}}(t), I(t), f(t,a)$ has now $\mu _b(t,a) = - \mu _0 a$, $D(a) = D$ and $\beta (a) = \beta _0 + \beta _1 a^2$. In this framework, the stationary (i.e. time independent) distribution $f_\infty$ of (12) is given by the Gaussian probability distribution truncated over ${\mathbb {R}}^+$

$$\begin{aligned} f_\infty (a) = \exp ( - \alpha (a) ), \ \ \hbox {where} \ \ \alpha (a) = \frac{\mu _0 a^2}{2D} + \frac{1}{2} \log \frac{\pi D}{2 \mu _0}, \end{aligned}$$

that is,

$$\begin{aligned} f_\infty (a) = \sqrt{\frac{2 \mu _0}{\pi D}}\exp \left( -\frac{\mu _0 a^2}{2D}\right) \end{aligned}$$

Since $\alpha ''(a) = \mu _0/D$ the logarithmic Sobolev inequality (16) writes in this case:

23 $$\begin{aligned} \int _{{\mathbb {R}}^+} f(a) \psi (a) \, da \le \frac{D}{2 \mu _0} \int _{{\mathbb {R}}^+} f(a) \left| \frac{d \psi (a)}{da} \right| ^2 da, \ \ \hbox {where} \ \ \psi = \log \frac{f}{f_\infty }. \end{aligned}$$

The statement of Theorem 2 in this case writes with a further result about convergence of the average transmission rate.

#### Theorem 4

Under Assumptions (i), (ii) and (iii), $t \mapsto I(t)$ converges to 0 and $t \mapsto {\overline{S}}(t)$ converges to ${\overline{S}}_\infty$ as time *t* goes to $+\infty$ for some ${\overline{S}}_\infty$ such that $0\le {\overline{S}}_\infty < {\overline{S}}(0)$. Moreover, as soon as $f_0 \log (f_0/f_\infty ) \in L^1({\mathbb {R}}^+)$, there exists $C_*>0$ such that for all $t>0$

$$\begin{aligned}{} & {} \left( \int _{{\mathbb {R}}^+} \left| f(t,a) - f_\infty (a)\right| da \right) ^2 \le C_* \int _0^t \frac{I(s)}{N} \exp (2 \mu _0(s-t)) \, ds \\{} & {} \qquad \qquad + 2 \exp (-2 \mu _0 t) \int _{{\mathbb {R}}^+} f_0(a) \log \frac{ f_0(a)}{f_\infty (a)} da, \end{aligned}$$

Moreover, the average transmission rate ${\overline{\beta }}(t)$ converges to

$$\begin{aligned} {\overline{\beta }}_\infty = \int _{{\textbf{R}}^+} \beta (a) f_\infty (a)\, da = \beta _0 +D \beta _1/\mu _0, \end{aligned}$$

as *t* goes to $+\infty$.

Under Assumptions (i), (ii) and (iii), the requirements (*H*1) (*H*2) (*H*3) and (*H*4) are satisfied. Therefore, Theorem 4 is contained in Theorem 2 above. Nonetheless, we provide here a detailed proof of Theorem 4 since it is simpler. It follows the lines of the proof of Theorem 2 with some algebraic and technical simplifications that make the whole argument more transparent. It also yields a stronger results regarding the convergence of the average transmission rate.

#### Proof

**Step 1: Convergence of** $({\overline{S}}(t), I(t))$

This is unchanged with respect to the previous proof.

**Step 2: Dynamics of** $t \mapsto {\overline{\beta }}(t)$

The evolution equation of *f* multiplied by $\beta (a)$ yields after integration over *a* variable:

$$\begin{aligned} \frac{d {\overline{\beta }}(t)}{dt} = - \frac{I(t)}{N} \hbox {Var } \beta (t) - \int _{{\mathbb {R}}^+} 2 \beta _1 a \left( \mu _0 a f(t,a) + D \frac{\partial f(t,a)}{\partial a} \right) da, \end{aligned}$$

where

$$\begin{aligned} \hbox {Var } \beta (t) = \int _{{\mathbb {R}}^+} \left( \beta (a) - {\overline{\beta }}(t)\right) ^2 f(t,a) \, da \ge 0. \end{aligned}$$

Then, introducing ${\overline{\beta }}_\infty = \beta _0 +D \beta _1/\mu _0$, using the results of Appendix C and integration by parts lead to

$$\begin{aligned} \frac{d {\overline{\beta }}(t)}{dt} + \frac{I(t)}{N} \hbox {Var } \beta (t)= & {} \int _{{\mathbb {R}}^+}\left( 2 \mu _0 (\beta _0 -\beta (a)) + 2 \beta _1 D \right) f(t,a) \, da. \\= & {} 2 \mu _0 \left( {\overline{\beta }}_\infty - {\overline{\beta }}(t) \right) . \qquad \qquad \qquad \qquad \end{aligned}$$

It follows that

$$\begin{aligned} {\overline{\beta }}(t) - {\overline{\beta }}_\infty \le \left( {\overline{\beta }}(0) - {\overline{\beta }}_\infty \right) \exp ( - 2 \mu _0 t ), \end{aligned}$$

so that ${\overline{\beta }}(t)$ is bounded and $\limsup _{t \rightarrow + \infty } {\overline{\beta }}(t) \le {\overline{\beta }}_\infty$.

**Step 3: Entropy estimate**

As before, we write the evolution equation of *f* in terms of $f_\infty$ and $\psi = \log (f / f_\infty )$:

$$\begin{aligned} \frac{\partial f(t,a)}{\partial t} = D \frac{\partial }{\partial a} \left( f(t,a) \frac{\partial \psi (t,a)}{\partial a} \right) - \left( \beta (a) - {\overline{\beta }}(t) \right) f(t,a) \frac{I(t)}{N}, \end{aligned}$$

so that multiplying the above equation by $\psi$ leads to

$$\begin{aligned} \frac{\partial }{\partial t} \left( f(t,a) \psi (t,a) - f (t,a) \right)= & {} D \frac{\partial }{\partial a} \left( f(t,a) \psi (t,a) \frac{\partial \psi (t,a)}{\partial a} \right) \\{} & {} - D f(t,a) \left| \frac{\partial \psi (t,a)}{\partial a}\right| ^2 \\{} & {} - \left( \beta (a) - {\overline{\beta }}(t) \right) f(t,a) \psi (t,a) \frac{I(t)}{N}. \end{aligned}$$

Integrating over ${\mathbb {R}}^+$, using the homogeneous Neumann boundary conditions on $\psi$, and introducing the non-negative function $\xi$ defined for positive *x* by $\xi (x)=x \log x - x +1$, we obtain

$$\begin{aligned} \frac{d}{dt} \int _{{\mathbb {R}}^+} f(t,a) \psi (t,a) \,da= & {} \left[ D f(t,a) \psi (t,a) \frac{\partial \psi (t,a)}{\partial a} \right] _0^{+\infty } \\{} & {} -\, D \int _{{\mathbb {R}}^+} f(t,a) \left| \frac{\partial \psi (t,a)}{\partial a}\right| ^2 da \\{} & {} -\, \frac{I(t)}{N} \int _{{\mathbb {R}}^+} \beta (a) f_\infty (a) \frac{f(t,a)}{f_\infty (a)} \log \frac{f(t,a)}{f_\infty (a)} da \\{} & {} + \frac{I(t)}{N} \int _{{\mathbb {R}}^+} \beta (a) f_\infty (a) \left( \frac{f(t,a)}{f_\infty (a)} - 1 \right) da \\{} & {} +\, \frac{I(t)}{N} \left( {\overline{\beta }}_\infty - {\overline{\beta }}(t) \right) + \frac{I(t)}{N} {\overline{\beta }}(t) \int _{{\mathbb {R}}^+} f(t,a) \psi (t,a) \,da. \end{aligned}$$

The first term of the right hand side vanishes: for $a=0$ because of the homogeneous Neumann boundary condition on $\psi (t,\cdot ) = f(t,\cdot ) / f_\infty$, and for $a=+\infty$ because $f(t,\cdot )$ decays like $f_\infty$ as proved in Appendix C. We therefore end up with

24 $$\begin{aligned}{} & {} \frac{d}{dt} \int _{{\mathbb {R}}^+} f(t,a) \psi (t,a) \,da + D \int _{{\mathbb {R}}^+} f(t,a) \left| \frac{\partial \psi (t,a)}{\partial a}\right| ^2 da \nonumber \\{} & {} \qquad + \frac{I(t)}{N} \!\!\int _{{\mathbb {R}}^+} \!\!\!\!\beta (a) \xi \left( \frac{f(t,a)}{f_\infty (a)}\right) f_\infty (a) \, da \nonumber \\{} & {} \quad = \frac{I(t)}{N} \left( {\overline{\beta }}_\infty - {\overline{\beta }}(t) \right) + \frac{I(t)}{N} {\overline{\beta }}(t) \int _{{\mathbb {R}}^+} f(t,a) \psi (t,a) \, da . \end{aligned}$$

Then, denoting the relative entropy

25 $$\begin{aligned} {{\mathcal {E}}}(t) = \int _{{\mathbb {R}}^+} f(t,a) \psi (t,a) \, da, \end{aligned}$$

we deduce that

$$\begin{aligned} \frac{d {{\mathcal {E}}}(t)}{dt} + D \int _{{\mathbb {R}}^+} f(t,a) \left| \frac{\partial \psi (t,a)}{\partial a}\right| ^2 da \le \frac{I(t)}{N} \left( {\overline{\beta }}_\infty + {\overline{\beta }}(t) {{\mathcal {E}}}(t) \right) . \end{aligned}$$

Finally, using the logarithmic Sobolev inequality (23), we obtain

$$\begin{aligned} \frac{d {{\mathcal {E}}}(t)}{dt} + 2 \mu _0 {{\mathcal {E}}}(t) \le \frac{I(t)}{N} \left( {\overline{\beta }}_\infty + {\overline{\beta }}(t) {{\mathcal {E}}}(t) \right) . \end{aligned}$$

Gronwall type lemma then leads to

$$\begin{aligned} {{\mathcal {E}}}(t) \le C_* \int _0^t \frac{I(s)}{N} \exp ( - 2 \mu _0 (s-t)) \, ds + {{\mathcal {E}}}(0) \exp ( - 2 \mu _0 t), \end{aligned}$$

for some positive constant $C_*$. Estimate (4) then follows from Csiszár-Kullback-Pinsker’s inequality (6) up to a change of $C_*$ by a factor 2.

**Step 4: Convergence of** ${\overline{\beta }}(t)$ to ${\overline{\beta }}_\infty$

We have obtained the $L^1$ convergence of $f(t,\cdot )$ to $f_\infty$, so that application of Fatou’s lemma leads to

$$\begin{aligned} \int _{{\mathbb {R}}^+} \liminf _{t \rightarrow + \infty } \beta (a) f(t,a)\, da \le \liminf _{t \rightarrow + \infty } \int _{{\mathbb {R}}^+} \beta (a) f(t,a)\, da, \end{aligned}$$

which means that $\liminf _{t \rightarrow + \infty } {\overline{\beta }}(t) \ge {\overline{\beta }}_\infty$. Since $\limsup _{t \rightarrow + \infty } {\overline{\beta }}(t) \le {\overline{\beta }}_\infty$ as established above, we conclude that ${\overline{\beta }}(t)$ converges to ${\overline{\beta }}_\infty$. $\square$

The simpler assumptions above also turn out to exhibit interesting properties regarding self-similar solutions. Such a viewpoint is developed in Sects. 4 and 6.

## The parsimonious model in the case of a Gaussian distribution

In order to obtain a parsimonious model, i.e. with as few parameters as possible, we look for self-similar solutions of (11). Dimarco et al. (2021) introduced a similar approach combining behavioral heterogeneity with social diffusion in the framework of kinetic theory and using gamma-type distributions of the susceptible population. Heterogeneity of susceptibles is described by a trait $b \in {\mathbb {R}}^+$ with transmission rate expressed as power laws of *b*.

In this section, we focus on Gaussian distribution profiles truncated on $A = {\mathbb {R}}^+$, which as explained later is connected to the Dimarco et al. (2021) modeling approach.

### Assumptions

In order to compute the reduced complexity model arising from self-similar solutions, we make the following assumptions:

- *Truncated Gaussian distribution* The initial distribution $f_0(a) = S_0(a) / {\overline{S}}_0$ of the susceptible population is described in terms of the continuous risk variable $a \in \mathbb {R^+}$
  $$\begin{aligned} f_0(a) = \frac{1}{\uplambda _0} \phi \left( \frac{a}{\uplambda _0}\right) \ \ \hbox {where} \ \ \uplambda _0 >0 \ \ \hbox {and} \ \ \phi (a) = \sqrt{\frac{2}{\pi }} \exp \left( - \frac{a^2}{2}\right) . \end{aligned}$$
  The approach in Dimarco et al. (2021) consists in considering Maxwellians in the kinetic theory framework expressed as gamma type distributions. The two approaches are connected through a change of variables in the probability measures: introducing trait *b* as $b=a^2/2$, one has
  $$\begin{aligned} \phi (a) da = (\pi b)^{-1/2} \exp (-b) db, \end{aligned}$$
  which is nothing but a gamma distribution with parameter 1/2. Let us also observe that the expression as truncated Gaussian distributions allows to derive more general models representing solutions of the original PDE model (11) in a more accurate way. The basic idea is to expand solutions $(t,a) \mapsto S(t,a)$ along even eigenfunctions of the underlying linear operator which turns out to coincide with the Schrödinger operator associated with harmonic oscillator potential. We provide a detailed derivation of the corresponding high order model in Appendix B, Sect. 6, together with numerical simulations illustrating the convergence of high order model to solutions of the full partial differential equation system.
- Constant diffusion coefficient with respect to *a*, possibly time dependent
  $$\begin{aligned} \frac{\sigma ^2 }{2} = D(t) \quad \hbox {for some positive function} \quad t \mapsto D(t). \end{aligned}$$
- The transmission rate function $\beta$ is a quadratic function of *a*
  $$\begin{aligned} \beta (a) = \beta _0 + \beta _1 a^2. \end{aligned}$$
  Note that this expression differs from Dimarco et al. (2021), which does not include the constant coefficient $\beta _0$.
- Background drift effect
  $$\begin{aligned} \mu _b (t,a) = - \mu _0(t) a . \end{aligned}$$
  Note that one may replace $\mu _0(t)$ by any function of time only, related or not to epidemic variables (it may be used for instance to account for the influence of public health policy).
- The susceptible population *S*(*t*, *a*) is assumed to be expressed as a self-similar profile
  26 $$\begin{aligned} S(t,a) = \frac{{\overline{S}}(t)}{\uplambda (t)} \phi \left( \frac{a}{\uplambda (t)} \right) , \end{aligned}$$
  for some functions of time $t \mapsto {\overline{S}}(t)$ and $t \mapsto \uplambda (t)$ such that ${\overline{S}}(0)={\overline{S}}_0$ and $\uplambda (0)=\uplambda _0$.

Let us observe at this stage that $\phi '(A) = -A \phi (A)$ and $\phi ''(A) = (A^2 -1) \phi (A)$, which will be used in computations below.

### Derivation

We deduce from the first equation of (11) that

$$\begin{aligned} \left( \frac{{\dot{{\overline{S}}}}}{\uplambda } - \frac{{{\dot{\uplambda }}} {\overline{S}}}{\uplambda ^2} \right) \phi (A) - A \phi '(A) \frac{{{\dot{\uplambda }}} {\overline{S}}}{\uplambda ^2}= & {} - \frac{I \left( \beta _0 + \beta _1\uplambda ^2 A^2 \right) {\overline{S}}}{N \uplambda } \phi (A) \\{} & {} +\, \left( - \frac{\mu _0 {\overline{S}}}{ \uplambda } + \frac{D {\overline{S}}}{\uplambda ^3 } \right) \left( A^2 - 1 \right) \phi (A), \end{aligned}$$

hence

$$\begin{aligned}{} & {} \phi (A) \left[ \left( \frac{{\dot{{\overline{S}}}}}{\uplambda } - \frac{{{\dot{\uplambda }}} {\overline{S}}}{\uplambda ^2} \right) + \frac{I \beta _0 {\overline{S}}}{N \uplambda } - \frac{\mu _0 {\overline{S}}}{ \uplambda } + \frac{D {\overline{S}}}{\uplambda ^3 } \right] \\{} & {} \qquad \qquad = A^2 \phi (A) \left[ - \frac{{{\dot{\uplambda }}} {\overline{S}}}{\uplambda ^2} - \frac{I \beta _1\uplambda ^2 {\overline{S}}}{N \uplambda } - \frac{\mu _0 {\overline{S}}}{\uplambda ^2 } + \frac{D {\overline{S}}}{\uplambda ^3 } \right] \end{aligned}$$

We therefore require that the two time dependent functions on the left and right hand side of (27) are equal to zero:

$$\begin{aligned}{} & {} \frac{{{\dot{\uplambda }}}}{\uplambda } = - \frac{I \beta _1\uplambda ^2}{N } - \mu _0 + \frac{D}{\uplambda ^2 } \\{} & {} \left( \frac{{\dot{{\overline{S}}}}}{\uplambda } - \frac{{{\dot{\uplambda }}} {\overline{S}}}{\uplambda ^2} \right) + \frac{I \beta _0 {\overline{S}}}{N \uplambda } - \frac{\mu _0 {\overline{S}}}{\uplambda } + \frac{D {\overline{S}}}{\uplambda ^3 } = 0 \end{aligned}$$

hence substituting (27) into (27), one gets on the one hand

$$\begin{aligned} \frac{{\dot{{\overline{S}}}}}{{\overline{S}}} = - \frac{I \left( \beta _0 + \beta _1 \uplambda ^2\right) }{N}. \end{aligned}$$

On the other hand, the second equation of (11) leads to

$$\begin{aligned} \frac{dI}{dt} = I \left( \frac{{\overline{S}}( \beta _0 + \beta _1 \uplambda ^2 )}{N} - \gamma \right) \end{aligned}$$

As a consequence, the derived model writes as follows

27 $$\begin{aligned} \boxed { \left\{ \begin{aligned} \displaystyle \frac{d{{\overline{S}}}}{dt }&= \displaystyle - \frac{{\overline{S}}I \left( \beta _0 + \beta _1 \uplambda ^2\right) }{N} \\ \displaystyle \frac{d \uplambda }{dt}&= \displaystyle - \frac{I \beta _1\uplambda ^3}{N} - \mu _0 \uplambda + \frac{D}{\uplambda } \\ \displaystyle \frac{d I}{dt}&= \displaystyle \frac{{\overline{S}}I ( \beta _0 + \beta _1 \uplambda ^2 )}{N} - \gamma I \\ \displaystyle \frac{dR}{dt}&= \gamma I, \end{aligned} \right. } \end{aligned}$$

with initial conditions ${\overline{S}}(0) = {\overline{S}}_0$, $\uplambda (0)=\uplambda _0$, $I(0)= I_0$ and $R(0)=0$. Let us observe that the average of the risk parameter *a* is linked to the $\uplambda$ parameter:

$$\begin{aligned} {{\bar{a}}} (t) = \sqrt{\frac{2}{\pi }} \uplambda (t) \qquad \hbox {Var}(a) (t) = \uplambda (t)^2 \left( 1 - \frac{2}{\pi } \right) . \end{aligned}$$

### Dynamics of the average transmission rate ${\overline{\beta }}$

Let us recall the equation governing the probability density function $f(t,a)=S(t,a)/{\overline{S}}(t)$ of susceptible individuals

$$\begin{aligned} \partial _t f(t,a) = - \frac{I(t)}{N} f(t,a) \left( \beta (a) - {\overline{\beta }}(t) \right) + \mu _0 \frac{\partial }{\partial a} ( a f(t,a)) + \frac{\partial ^2}{\partial a^2} (D f (t,a)). \end{aligned}$$

Then, we introduce the notation

$$\begin{aligned} {\overline{\varphi }} (t) = \int _{{\mathbb {R}}^+} \varphi (a) f(t,a) \, da, \end{aligned}$$

so that one has

$$\begin{aligned} \frac{d {\overline{\beta }}(t)}{dt}= & {} - \frac{I(t)}{N} (\overline{\beta ^2}(t) - {\overline{\beta }}(t)^2) - \mu _0 \!\int _{{\mathbb {R}}^+} \beta '(a) a f (t,a) \, da - D \int _{{\mathbb {R}}^+} \!\!\beta '(a) \frac{\partial f (t,a)}{\partial a} \, da \\ {\overline{\beta }}(t)= & {} \int _{{\mathbb {R}}^+} \beta (a) f(t,a) \, da \\ Var(\beta )(t)= & {} \overline{\beta ^2}(t) - {\overline{\beta }}(t)^2. \end{aligned}$$

Then, inserting the self similar profiles into (27), $f(t,a) = \uplambda (t)^{-1} \phi (a/\uplambda (t))$, one gets

$$\begin{aligned} {\overline{\beta }}(t)= & {} \beta _0 + \beta _1 \uplambda ^2. \\ \overline{\beta ^2}(t)= & {} \int _{{\mathbb {R}}^+} (\beta _0^2 + 2 \beta _0 \beta _1 \uplambda ^2 A^2 + \uplambda ^4 \beta _1^2 A^4) \phi (A) dA = \beta _0^2 + 2 \beta _0 \beta _1 \uplambda ^2 + 3 \beta _1^2 \uplambda ^4, \end{aligned}$$

where we used the fact that $A^2 \phi = \phi {''} + \phi$ and $A^4 \phi = ( A^2 \phi ' + 3 \phi ')^{'} + 3 \phi$. The variance $\hbox {Var}(\beta )(t)$ can then be expressed as

$$\begin{aligned} \hbox {Var}(\beta )(t) = 2 \beta _1^2 \uplambda ^4(t) = 2 ({\overline{\beta }}- \beta _0)^2 \end{aligned}$$

It means that the dynamics of $t \mapsto \uplambda (t)$ is directly linked to the average value of the $\beta$ parameter. Therefore, the equation on $\uplambda$ can be replaced by the dynamics of the average transmission rate ${\overline{\beta }}$ in System (27)

$$\begin{aligned} \frac{d {\overline{\beta }}}{dt} =- 2 \frac{I}{N} ({\overline{\beta }}- \beta _0)^2 - 2 \mu _0 ({\overline{\beta }}- \beta _0) + 2 D \beta _1. \end{aligned}$$

Therefore, (27) becomes

27 $$\begin{aligned} \boxed { \left\{ \begin{aligned} \displaystyle \frac{d{{\overline{S}}}}{dt }&= \displaystyle - \frac{{\overline{\beta }}\, {\overline{S}}\, I }{N} \\ \displaystyle \frac{d {\overline{\beta }}}{dt}&= \displaystyle - 2 \frac{I}{N} ({\overline{\beta }}- \beta _0)^2 - 2 \mu _0 ({\overline{\beta }}- \beta _0) + 2 D \beta _1 \\ \displaystyle \frac{d I}{dt}&= \displaystyle \frac{ {\overline{\beta }}\, {\overline{S}}\, I}{N} - \gamma I \\ \displaystyle \frac{dR}{dt}&= \gamma I, \end{aligned} \right. } \end{aligned}$$

with initial conditions $I(0) = I_0 \in (0,N)$, ${\overline{S}}(0) \in (0,N-I_0]$, $R(0)=N-I(0)-S(0)$ and ${\overline{\beta }}(0) = {\overline{\beta }}_0 \ge \beta _0$.

We have thus reduced the PDE system (11) to an ODE system with four unknowns. It is worth emphasizing that this ODE system, albeit simple, retains the memory of the PDE system, through the diffusion *D* and the drift $\mu _0$.

This system is a simple extension of the *SIR* model involving a parameter for social diffusion *D* and an additional equation for the dynamics of the average transmission rate ${\overline{\beta }}(t)$. This last effect is indeed a major characteristic of the evolution of the epidemics. Indeed, from the second equation in System (27), we see that, on the one hand, ${\overline{\beta }}(t)$ has a tendency to decrease under the effect of the epidemics sculpting the distribution of *a* by removing relatively more high *a* values, while, on the other hand, the social diffusion parameter tends to offset this tendency.

### A rich variety of dynamical behaviors

We now show that this reduced form still yields a wealth of various dynamical behaviors. In fact, we recover nearly all the dynamical features we reported in our earlier work (Berestycki et al. 2021) for the full PDE model (11). All the figures we show here are obtained from numerical simulations of System (27) over a period of 18 months with realistic values of epidemic parameters, initialized with a very small sub-population of infected individuals in an otherwise susceptible population.

**Fig. 1:**
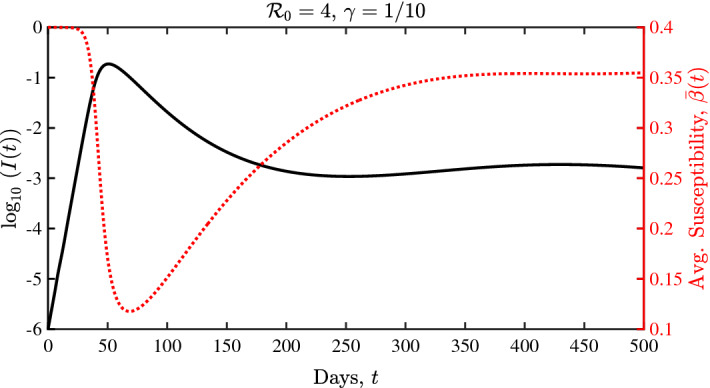
Epidemic dynamics leading to a single peak followed by a shoulder. Dynamics shown are that of infected fraction, *I*(*t*) (black, solid) in logarithmic scale, and Average susceptibility, ${\overline{\beta }}(t)$ (red, dashed). Parameters are ${\overline{\beta }}(0)=0.4 \, \hbox {days}^{-1}$, $\beta _0=0\, \hbox {days}^{-1}$, $\mu _0=5\times 10^{-3}\, \hbox {day}^{-1}$, $\gamma =0.1\, \hbox {days}^{-1}$, $2D\beta _1 = 0.004\, \hbox {days}^{-2}$, ${{\mathcal {R}}}_0=4$, $I(0)=10^{-6}$

In Fig. 1, we consider the case in which ${{\mathcal {R}}}=4$ given an average infectious period of 10 days (full parameters included in the caption). The initial value of *f* is set to the equilibrium value expected in the absence of an epidemic. We then evaluate dynamics given an initial infected population fraction of $10^{-6}$. We observe an exponential growth, followed by a decay and then a smaller shoulder pattern. This is a representative sequence observed in epidemic data. Note that during the exponential growth phase, the average susceptibility remains close to the equilibrium level. However, when a sufficiently large number of individuals are infected, then the average susceptibility drops, leading to a decrease in susceptibility which then relaxes over time, enabling the re-emergence of a longer term shoulder. Over time, the infections decay and the susceptibility relaxes slowly towards ${\overline{\beta }}^{*}$ (over a multi-year period given this parameter set). We emphasize that, though much simplified with respect to the full PDE model, this system of ODEs can generate complex features.

**Fig. 2:**
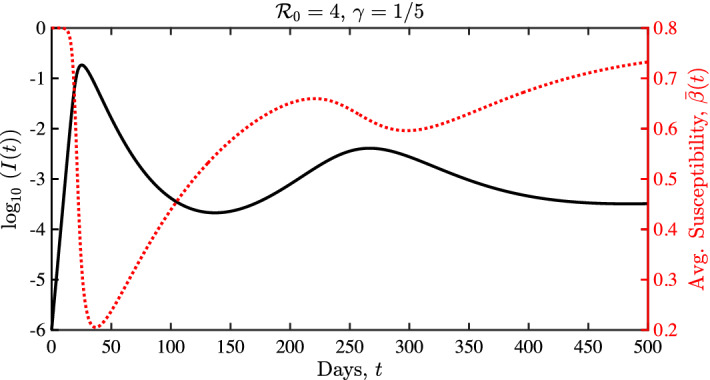
Epidemic dynamics leading to multiple peaks. Dynamics shown are that of infected fraction, *I*(*t*) (black, solid) in logarithmic scale, and Average susceptibility, ${\overline{\beta }}(t)$ (red, dashed). Parameters are: ${\overline{\beta }}(0)=0.8\, \hbox {day}^{-1}$, $\beta _0=0.0\, \hbox {day}^{-1}$, $I(0)=1E-6$, $2D\beta _1 = 0.008\, \hbox {day}^{-2}$, $\mu _0=5E-3\, \hbox {day}^{-1}$, $\gamma =0.2\, \hbox {day}^{-1}$

As we already pointed out in our previous article (Berestycki et al. 2021) rebounds appear as part of the *intrinsic* dynamics of the epidemics even without variants. This is illustrated in Fig. 2 with larger amplitudes of oscillations. In this case however, as should be expected, further rebounds reach lower maximal levels than the first peak because of the dissipative nature of the underlying PDE model. As before, the ${\overline{\beta }}(t)$ decreases as infections increase and then revert back towards the equilibrium, enabling the second oscillation. The initial decrease in susceptibility reflects how the ODE retains the sculpting feature of the PDE while the rebound is a result of the drift back to the mean of the unique equilibrium distribution in *f*.

Note that for suitable parameters System (27) also exhibits the typical SIR behavior of an exponential growth followed by a (nearly) exponential decay. We perform more systematic comparison of PDE and ODE based dynamics in Sect. 6.1.

## Mathematical analysis of the ODE system

### Properties of the ODE system

This section is devoted to deriving several mathematical properties of the ODE system (27). Our purpose here is to show that this reduced system displays natural features of the heterogeneous system. We mainly discuss here the large time behavior of $({\overline{S}}(t), {\overline{\beta }}(t), I(t), R(t))$ in terms of the parameters $\mu _0$, *D* and ${\overline{\beta }}(0)$. The results and their dependence on the parameters are summarized in Table 1 at the end of the subsection.

Throughout this section, without loss of generality, we normalize the population in such a way that $N=1$.

#### Consistency with *SIR* model

We first observe that (27) reduces to the standard *SIR* model in the absence of heterogeneity i.e. $\beta _1=0$, when ${\overline{\beta }}(0) = \beta _0$. Clearly, in this case there holds ${\overline{\beta }}(t) = \beta _0$ for all $t\ge 0$ and we get the solution of the corresponding *SIR* system.

#### Bounds on the average transmission rate ${\overline{\beta }}$

We claim that the following proposition holds

##### Proposition 1

Assume that ${\overline{\beta }}(0) \ge \beta _0$, $\mu _0\ge 0$ and $D \ge 0$. Then, for all $t\ge 0$, one has ${\overline{\beta }}(t) \ge \beta _0$.

##### Proof

We argue by contradiction. In the case $D>0$, suppose there is a first time $t_*$ where ${\overline{\beta }}(t_*)=\beta _0$. Then, from the equation we get ${\overline{\beta }}'(t_*) >0$ which is a contradiction. In the case $D=0$, the equation for $x={\overline{\beta }}-\beta _0$ is of the form $\dot{x} (t)= g(t,x)x(t)$, so that $x(t) >0$ when $x(0) >0$. $\square$

#### Asymptotic limit of $({\overline{S}},I,R)$

As in the conventional *SIR* model, the solution converges to a constant state in the large time limit.

##### Proposition 2

The solution of (27) $({\overline{S}}(t),I(t),R(t))$ converges to $({\overline{S}}_\infty ,0,R_\infty )$ as *t* goes to $+\infty$, with ${\overline{S}}_\infty \in [0,1)$, $R_\infty \in (0,1]$ such that ${\overline{S}}_\infty +R_\infty =1$.

##### Proof

$t \mapsto R(t)$ is non-decreasing and bounded by 1, so that $R(t) \rightarrow R_\infty \in (0, 1]$ as $t \rightarrow +\infty$. Moreover, $t \mapsto {\overline{S}}(t)$ is non-increasing and positive, so that ${\overline{S}}(t) \rightarrow {\overline{S}}_\infty$ for some ${\overline{S}}_\infty \in [0,1)$. It follows that $I(t) = 1 - {\overline{S}}(t) - R(t)$ converges to some $I_\infty \ge 0$. Since *R*(*t*) converges to some finite limit $R_\infty$, one has $I_\infty =0$. $\square$

#### The case $\mu _0>0$

##### Proposition 3

In the case when $\mu _0>0$, the average transmission coefficient ${\overline{\beta }}(t)$ remains bounded and converges to ${\overline{\beta }}_\infty = \beta _0+D \beta _1/\mu _0$ as *t* goes to $+\infty$. Moreover, the limits satisfy

28 $$\begin{aligned} {\overline{\beta }}_\infty {\overline{S}}_\infty \le \gamma . \end{aligned}$$

##### Proof

First one has

$$\begin{aligned} \frac{d {\overline{\beta }}}{dt} \le - 2 \mu _0 ({\overline{\beta }}- \beta _0) + 2 D \beta _1, \end{aligned}$$

so that $t \mapsto {\overline{\beta }}(t)$ satisfies

$$\begin{aligned} {\overline{\beta }}(t) \le \beta _0 + ({\overline{\beta }}(0) - \beta _0) \exp ( - 2\mu _0 t ) + \frac{D \beta _1}{ \mu _0} \left( 1 - \exp ( - 2\mu _0 t ) \right) \end{aligned}$$

and thus remains bounded: there exists $M>0$ such that ${\overline{\beta }}(t) \le M$ for all $t \in {\mathbb {R}}^+$. Then, taking ${\overline{\beta }}_\infty$ as defined in the above proposition, one also has

29 $$\begin{aligned} {\overline{\beta }}(t) - {\overline{\beta }}_\infty= & {} ({\overline{\beta }}(0) - {\overline{\beta }}_\infty ) \exp ( - 2\mu _0 t )\nonumber \\{} & {} - \,\int _0^t \!2 ({\overline{\beta }}(s) - \beta _0)^2 I(s) \exp ( -2 \mu _0 (t-s)) \, ds. \end{aligned}$$

Given $\varepsilon >0$, for *T* large enough, and $t>T$ one has

$$\begin{aligned} \int _T^t 2 ({\overline{\beta }}(s) - \beta _0)^2 I(s) \exp ( -2 \mu _0 (t-s)) \, ds \le 2 M^2 \int _T^{+\infty } \!\!I(s)\, ds \le \varepsilon /2. \end{aligned}$$

Hence, taking *T* large enough and, say, $t> 2T$, we see that the right hand side of (29) is less than $\varepsilon$.

This proves the convergence of ${\overline{\beta }}(t)$ to ${\overline{\beta }}_\infty$ as $t\rightarrow +\infty$. Finally, in view of the equation satisfied by *I*, it is straightforward to see that inequality (28) holds. $\square$

Notice that ${\overline{\beta }}(t)$ converges to the same limit ${\overline{\beta }}_\infty$ as in the case of solutions of the full PDE system (12) in the Gaussian case (compare the above Theorem 4).

It is interesting to observe that if initially $I(0) = I_0 >0$, and ${\overline{\beta }}(0) = {\overline{\beta }}_\infty$, then $t \mapsto {\overline{\beta }}(t)$ is decreasing in the early stages of the epidemic. Indeed, one has

$$\begin{aligned} \frac{d {\overline{\beta }}}{dt} (0) = - 2 I_0 ({\overline{\beta }}(0) - \beta _0)^2 = - \frac{2 I_0 D^2 \beta _1^2}{\mu _0^2 } < 0. \end{aligned}$$

Thus, if initially the epidemic depletes more the fraction of the population with large *a*, eventually, for large time, this trend will be reversed and ${\overline{\beta }}(t)$ will increase to converge to its equilibrium value ${\overline{\beta }}_\infty$. We also established this property in the full PDE model (compare Theorem 4).

#### The case $\mu _0=0$ and $D>0$

In the case when $\mu _0=0$ and $D>0$, the following properties hold

##### Proposition 4

In the case when $\mu _0=0$ and $D>0$, the limit susceptibles satisfy ${\overline{S}}_\infty = 0$, $R_\infty = 1$, and ${\overline{\beta }}(t) \rightarrow + \infty$ as *t* goes to $+\infty$.

   Let us observe that the case $\mu _0=0$ is singular, since in that case all susceptibles become infected at the end of the epidemic. This property is related to the fact that Model (27) derives from the underlying partial differential equation model (11) where the risk trait *a* covers the unbounded domain ${\mathbb {R}}^+$.

##### Proof

First, we observe that $\tau = 1/({\overline{\beta }}- \beta _0)$ satisfies

$$\begin{aligned} \frac{d \tau }{dt} = 2 I - 2D\beta _1 \tau ^2, \end{aligned}$$

so that

$$\begin{aligned} 2D\beta _1 \int _0^t \tau (s)^2 ds = 2 \int _0^t I(s)ds - \tau (t) + \tau (0). \end{aligned}$$

Using the fact that $\tau \ge 0$, the integral on the left hand side converges since *I* itself is integrable over ${\mathbb {R}}^+$. As a consequence, $\tau (t)$ converges to some finite value $\tau _\infty \ge 0$ as *t* goes to $+\infty$. The convergence of the integral of $\tau ^2$ requires that $\tau _\infty =0$, i.e. ${\overline{\beta }}(t)$ tends to $+\infty$ as *t* goes to $+\infty$. Finally, assuming that ${\overline{S}}_\infty >0$ the dynamics of *I* would be exponentially growing since ${\overline{\beta }}(t) {\overline{S}}(t) - \gamma$ would tend to $+\infty$, which does not make sense. We conclude that ${\overline{S}}_\infty =0$, so that $R_\infty = 1$. $\square$

#### The case $D=0$

We derive here comparisons with the classical *SIR* model. To this end, we introduce the notation $S^{SIR, \alpha }(t)$ for the solution of the *SIR* model with transmission rate $\beta =\alpha$ (constant) and a fixed parameter $\gamma$. We write $S^{SIR, \alpha }_\infty$ for the final size of the susceptible population.

In the case when $\mu _0> 0$ and $D=0$, the following properties hold

##### Proposition 5

When $D=0$, the average transmission rate $t \mapsto {\overline{\beta }}(t)$ is non-increasing and converges to $\beta _0$ as *t* goes to $+\infty$. Moreover, if ${\overline{\beta }}(0) {\overline{S}}_0 > \gamma$ and $I_0>0$, then $t \mapsto I(t)$ has a unique maximum as in the homogeneous SIR model. Finally, the limit susceptible populations satisfies

$$\begin{aligned} S^{SIR, {\overline{\beta }}(0) }_\infty \le {\overline{S}}_\infty \le S^{SIR, \beta _0 }_\infty . \end{aligned}$$

##### Proof

As in the case of the homogeneous *SIR* model, the dynamics of susceptibles ${\overline{S}}$ in terms of *R* instead of time leads to the expression

$$\begin{aligned} {\overline{S}}(R) = {\overline{S}}_0 \exp \left( - \frac{\displaystyle \int _{R_0}^R {\overline{\beta }}(\rho ) d\rho }{\gamma }\right) \quad \hbox {for} \ \ R \in [R_0, R_\infty ]. \end{aligned}$$

Here $R_\infty$ is the final size of the *R*-population. It is given by the relation:

$$\begin{aligned} R_\infty + {\overline{S}}_0 \exp \left( - \frac{\displaystyle \int _{R_0}^{R_\infty } {\overline{\beta }}(\rho ) d\rho }{\gamma }\right) = 1. \end{aligned}$$

We introduce the notations:

$$\begin{aligned} F^\alpha (x) = x + {\overline{S}}_0 \exp \left( - \frac{\alpha (x-R_0) }{\gamma }\right) \quad \hbox {for all} \ \ x \in [R_0, +\infty ), \\ F(x) = x + {\overline{S}}_0 \exp \left( - \frac{\displaystyle \int _{R_0}^x {\overline{\beta }}(\rho ) d\rho }{\gamma }\right) \quad \hbox {for all} \ \ x\in [R_0, R_\infty ]. \end{aligned}$$

From the equation for ${\overline{\beta }}$ in System (27), we see that ${\overline{\beta }}(t)$ is decreasing (aside from the obvious case ${\overline{\beta }}(t)\equiv \beta _0$). Thus, in view of Proposition 1, we know that for all $t\ge 0$, one has ${\overline{\beta }}(0) \ge {\overline{\beta }}(t) \ge \beta _0$. Therefore, we infer that:

$$\begin{aligned} F^{{\overline{\beta }}(0)}(x) \le F(x)\le F^{\beta _0}(x) \quad \hbox {for all} \ \ x\in [R_0, R_\infty ]. \end{aligned}$$

From the classical final size relation in the *SIR* model, we deduce that

$$\begin{aligned} S^{SIR, {\overline{\beta }}(0) }_\infty \le {\overline{S}}_\infty \le S^{SIR, \beta _0 }_\infty . \end{aligned}$$

$$\square $$

#### Expression in terms of *R* in the general case $D\ge 0$

As in the case of *SIR* model, we observe that we can express all the variables ${\overline{S}}, {\overline{\beta }}, I$ in terms of *R*:

30 $$\begin{aligned} \left\{ \begin{aligned} \displaystyle \frac{d{{\overline{S}}}}{dR }&= \displaystyle - \frac{{\overline{\beta }}\, {\overline{S}}}{\gamma } \\ \displaystyle \frac{d {\overline{\beta }}}{dR}&= \displaystyle - \frac{2}{\gamma } ({\overline{\beta }}- \beta _0)^2 - \frac{2 \mu _0 ( {\overline{\beta }}- \beta _0)}{\gamma I} + \frac{2 D \beta _1}{\gamma I} \\ \displaystyle \frac{d I}{dR}&= \displaystyle \frac{ {\overline{\beta }}\, {\overline{S}}}{\gamma } - 1 . \end{aligned} \right. \end{aligned}$$

From the first equation of (30), we infer

$$\begin{aligned} {\overline{S}}(R) = {\overline{S}}_0 \exp \left( - \frac{B(R)}{\gamma } \right) , \quad \hbox {where} \quad B(R) = \int _{R_0}^R {\overline{\beta }}(\rho ) d \rho , \end{aligned}$$

so that the infected write as

$$\begin{aligned} I(R) = 1 - R - {\overline{S}}_0 \exp \left( - \frac{B(R)}{\gamma } \right) . \end{aligned}$$

The second equation then leads to

$$\begin{aligned} B''(R) = - \frac{2 (B'(R) - \beta _0)^2 }{\gamma } + \frac{2 D \beta _1 - 2 \mu _0 (B'(R) - \beta _0) }{\displaystyle \gamma \left( 1 - R - {\overline{S}}_0 \exp \left( - \frac{B(R)}{\gamma } \right) \right) }. \end{aligned}$$

#### Without social diffusion $D=0$ and without drift $\mu _0=0$

In the absence of social diffusion $D=0$ and drift $\mu _0=0$, the system describes a heterogeneous population without random fluctuations of the risk factor. It turns out that in this case, we can solve explicitly System (27) in terms of *R* to get:

31 $$\begin{aligned} B(R) = \beta _0 (R-R_0) + \frac{\gamma }{2} \log \left( 1 + \frac{2(R-R_0) ({\overline{\beta }}(0) -\beta _0)}{\gamma } \right) , \end{aligned}$$

i.e.

$$\begin{aligned} {\overline{\beta }}(R) = \beta _0 + \frac{{\overline{\beta }}(0) - \beta _0}{\displaystyle 1 + \frac{2(R-R_0)}{\gamma } ({\overline{\beta }}(0) - \beta _0) }. \end{aligned}$$

The infected then write in terms of *R* as

$$\begin{aligned} I(R) = 1 - R - \frac{\displaystyle {\overline{S}}_0 \exp \left( - \frac{\beta _0 (R-R_0)}{\gamma } \right) }{\displaystyle \left( 1 + \frac{2(R-R_0) ({\overline{\beta }}(0) -\beta _0)}{\gamma } \right) ^{1/2}}. \end{aligned}$$

When ${\overline{\beta }}(0)=\beta _0$, one recovers the classical formulas of the conventional *SIR* model. Furthermore, at the initial stage, when *R* is close to $R_0$, we get

$$\begin{aligned} I(R) \approx I_0 + (R-R_0) \left( \frac{{\overline{\beta }}(0) {\overline{S}}_0}{\gamma } -1 \right) . \end{aligned}$$

This formula generalizes the classical criterion $\mathcal {R}_0 >1$ for epidemic expansion. Here, it is expressed in terms of the parameter ${\mathcal {R}}_0={\overline{\beta }}(0) {\overline{S}}_0/\gamma$.

#### Entropy estimates when $\mu _0>0$ and $D>0$

Let us translate Estimate (24) of the relative entropy (25) in the framework of self-similar solutions as developed in Sect. 4. Denoting

$$\begin{aligned} \theta (t) = \frac{\uplambda (t)^2}{\uplambda _\infty ^2} \ \ \hbox {where} \ \ \uplambda _\infty = \sqrt{\frac{D}{\mu _0}}, \end{aligned}$$

we infer from self similar expression (26) of $f(t,\cdot )$ that

$$\begin{aligned} {{\mathcal {E}}}(t) = \int _ \mathbb {R^+} f(t,a) \log \frac{f (t,a)}{f_\infty (a)} da = \frac{1}{2} \left( \theta (t) - 1 - \log \theta (t) \right) , \end{aligned}$$

whereas its dissipation rate rewrites as the simple equation

$$\begin{aligned} {{\mathcal {D}}}(t) = D \int _{{\mathbb {R}}^+} f(t,a) \left| \frac{\partial \psi (t,a)}{\partial a}\right| ^2 da = \mu _0 \frac{ (1 - \theta (t))^2}{\theta (t)}. \end{aligned}$$

Then, Estimate (24) rewrites in terms of $\theta$ as

$$\begin{aligned} \frac{d {{\mathcal {E}}}}{dt} + {{\mathcal {D}}}(t) = 0 \end{aligned}$$

We observe that the Logarithmic Sobolev inequality is then associated to the property that for all $x \in {\mathbb {R}}^+$

$$\begin{aligned} 0 \le x - 1 - \log x \le \frac{(x-1)^2}{x}. \end{aligned}$$

Hence, we obtain the exponential convergence of the relative entropy

$$\begin{aligned} {{\mathcal {E}} }(t) \le {{\mathcal {E}} }(0) \exp ( - 2 \mu _0 t), \end{aligned}$$

which provides another proof of convergence of $\uplambda (t)$ to $\uplambda _\infty$ (hence proving the convergence of ${\overline{\beta }}(t)$ to ${\overline{\beta }}_\infty$).

We summarize the previous results in the following synthetic Table 1.

**Table 1:** Summary of large-time behavior of solutions of (27)

Assumptions			Large time properties	
$\mu _0$	*D*	${\overline{\beta }}(0)$	${\overline{\beta }}(t)$	$({\overline{S}}(t), I(t), R(t))$
$\ge 0$	$\ge 0$	$\ge \beta _0$	${\overline{\beta }}(t) \ge \beta _0$	$({\overline{S}}(t), I(t), R(t)) \rightarrow ({\overline{S}}_\infty , 0, R_\infty )$, ${\overline{S}}_\infty \in [0,1)$ and $R_\infty \in (0,1]$
$> 0$	$\ge 0$	$\ge \beta _0$	${\overline{\beta }}(t) \rightarrow {\overline{\beta }}_\infty$ with ${\overline{\beta }}_\infty \!=\! \beta _0\!+\!D\beta _1/\mu _0$	${\overline{\beta }}_\infty {\overline{S}}_\infty \le \gamma$
$$ = 0$$	$$ > 0$$	$$\ge \beta _0$$	$${\overline{\beta }}(t) \rightarrow +\infty $$	$${\overline{S}}_\infty = 0$$, $$R_\infty = 1$$
$$ = 0$$	$$ = 0$$	$$> \beta _0$$	$${\overline{\beta }}(t) \rightarrow {\overline{\beta }}_\infty > \beta _0$$	$$ {\overline{\beta }}_\infty = \beta _0 + \frac{\displaystyle {\overline{\beta }}(0) \!-\! \beta _0}{ \displaystyle 1 \!+\! 2 (R_\infty \!-\!R_0)({\overline{\beta }}(0) \!-\! \beta _0)/\gamma }$$
$$ > 0$$	$$ = 0$$	$$\ge \beta _0$$	$${\overline{\beta }}(t) \rightarrow \beta _0$$	$$ S_\infty ^{SIR, {\overline{\beta }}(0)} \le {\overline{S}}_\infty \le S_\infty ^{SIR, \beta _0}$$

The new systems we have introduced here beg for further study. These concern both the PDE system (12) and the ODE system (27). They lead to several open problems among which we mention the following three.

- What is the dependence of ${\overline{S}}_\infty$ in the PDE system (12) with respect to model parameters $\mu _0, D, \beta _1$ etc.?
- Derive the asymptotics for the PDE model (12) when $\mu _0 \rightarrow +\infty$. We conjecture that the probability distribution *f*(*t*, *a*) converges to a Dirac distribution $\delta _{a=0}$, and $\beta (t,a) \rightarrow \beta _0$ for any $t>0$. Therefore, in the limit, we should get the *SIR* model with $\beta _0$ as the transmission rate. The same question is also relevant for the ODE model.
- Derive the asymptotics when $D\rightarrow \infty$ in the ODE and PDE modeling frameworks. Here we conjecture that this situation should be analogous to the case $\mu _0=0$ and that ${\overline{\beta }}(t)\rightarrow \infty$, $I(t)\rightarrow 0$, ${\overline{S}}(t)\rightarrow 0$ for any fixed $t>0$.

### Oscillations: heuristic arguments

In the unfolding of epidemics, when there is a plateau, one often observes oscillatory behavior of the level of infected. Therefore, it is useful to check the ability of a model to reproduce this stylized fact. We provide here heuristic evidence that oscillations may indeed occur in the simplified model (27).

We only consider the case $\mu _0=0$, and $\beta _0=0$. As for other epidemic features, we find it more convenient to work in logarithmic scale, that is, with the variable $\log I$ rather than *I*. We seek some constant value $I_p$ that we will choose adequately later on, such that there is a plateau at the level $I\simeq I_p$. We set $X = \log ( I / I_p)$. Again, we assume that $N=1$ for simplicity. Introducing $\eta = 2 D \beta _1$, the system (27) takes the form:

$$\begin{aligned} \left\{ \begin{aligned} \displaystyle \frac{d{{\overline{S}}}}{dR }&= \displaystyle - \frac{{\overline{\beta }}\, {\overline{S}}}{\gamma } \\ \displaystyle \frac{d {\overline{\beta }}}{dR}&= \displaystyle - \frac{2{\overline{\beta }}^2}{\gamma } + \frac{\eta }{\gamma I} \\ \displaystyle \frac{d X}{dR}&= \displaystyle \left( \frac{ {\overline{\beta }}\, {\overline{S}}}{\gamma } - 1 \right) \frac{1}{I}. \end{aligned} \right. \end{aligned}$$

Hence, computing the second derivative of *X* with respect to *R* we get

$$\begin{aligned} \frac{d^2X}{dR^2 } = - \left( \frac{{\overline{\beta }}\, {\overline{S}}}{\gamma }- 1 \right) ^2 \frac{1}{I^2} + \frac{ {\overline{S}}}{\gamma ^2} \left( \frac{\eta }{I} - 3 {\overline{\beta }}^2 \right) \frac{1}{I}. \end{aligned}$$

Denoting $\rho = {\overline{\beta }}\, {\overline{S}}/ \gamma -1$, we reformulate the above ODE as follows

32 $$\begin{aligned} \frac{d^2X}{dR^2 } = \frac{1}{I^2} \left( - \rho ^2 + \frac{\eta {\overline{S}}}{\gamma ^2} \right) - \frac{3 (1+\rho )^2}{I {\overline{S}}}. \end{aligned}$$

In a plateau, one expects the right hand side of this equation to be very small. It is then natural to introduce the value of *I* that makes the right hand side vanish. We denote it by ${{\tilde{I}}}_p$:

$$\begin{aligned} {{\tilde{I}}}_p = \frac{\eta {\overline{S}}^2 - \rho ^2 \gamma ^2 {\overline{S}}}{3 \gamma ^2 (1+\rho )^2}. \end{aligned}$$

Note that ${{\tilde{I}}}_p$ varies in time like ${\overline{S}}$. Thus, Eq. (32) rewrites as

33 $$\begin{aligned} \frac{d^2X}{dR^2 } = \frac{3 (1+\rho )^2}{{\overline{S}}} \left( \frac{{{\tilde{I}}}_p}{I^2} - \frac{1}{I } \right) . \end{aligned}$$

The idea now is to seek a constant plateau value $I_p$ close to ${{\tilde{I}}}_p$ for which we can find oscillations around it. To this end, since $I=I_p\exp (X)$, it is convenient to reformulate Eq. (33) in the following manner:

$$\begin{aligned} \frac{d^2X}{dR^2 } = \frac{3 (1+\rho )^2}{{\overline{S}}} \left( \frac{{{\tilde{I}}}_p}{I_p^2}\exp (-2 X) - \frac{1}{I_p }\exp (-X) \right) . \end{aligned}$$

This ODE is of the form

$$\begin{aligned} \frac{d}{dR} \left( \begin{array}{c} X \\ \displaystyle \frac{dX}{dR} \end{array} \right) = M \cdot \left( \begin{array}{c} X \\ \displaystyle \frac{dX}{dR} \end{array} \right) + \left( \begin{array}{c} 0 \\ \displaystyle \frac{3 (1+\rho )^2}{{\overline{S}}I_p} \left( \frac{{{\tilde{I}}}_p}{I_p}-1 \right) \exp (-2X) \end{array} \right) \end{aligned}$$

where

$$\begin{aligned} M = \left( \begin{array}{cc} 0 &{} 1 \\ \displaystyle - \omega ^2 &{} 0 \end{array} \right) , \qquad \omega ^2 = \frac{3 (1+\rho )^2}{{\overline{S}}I_p} \left( \frac{\exp (-X) - \exp (-2X)}{X} \right) \end{aligned}$$

We then consider ranges of *R* such that ${{\tilde{I}}}_p \in (0,1)$ which requires that

$$\begin{aligned} 0< \eta {\overline{S}}^2 - \rho ^2 \gamma ^2 {\overline{S}}< 3 \gamma ^2 (1+\rho )^2. \end{aligned}$$

We interpret this relation as a necessary condition for a plateau to exist. Assume moreover that

$$\begin{aligned} | \rho | \ll 1 \end{aligned}$$

and $I_p$ is such that

$$\begin{aligned} \left| \frac{{{\tilde{I}}}_p}{I_p}-1 \right| \ll 1. \end{aligned}$$

Then, the complex eigenvalues of *M* are purely imaginary numbers which means that *I* oscillates around $I_p$ in logarithmic scale. Thus we find oscillations in this plateau regime.

For the above heuristic arguments to make sense, we surmise that in a plateau regime, ${\overline{S}}$ varies more slowly than the oscillations of *X*. Further mathematical developments need to be achieved to rigorously justify this reasoning.

## Higher order Gaussian based model

### Model derivation

An intermediate complexity model, which covers the case of the aforementioned Gaussian based model, can be derived using mathematical properties of the eigenvalues and eigenfunctions of the linear operator associated with Model (12). Incidentally, such operator coincides with the Schrödinger operator associated with the harmonic oscillator potential:

$$\begin{aligned} - \psi _n''(A) + A^2 \psi _n(A) = (2 n +1) \psi _n (A), \quad A \in {\mathbb {R}}. \end{aligned}$$

The eigenfunctions write in terms of the so called Hermite polynomials:

$$\begin{aligned} \psi _n(A) = H_n(A) \exp \left( - A^2/2 \right) / \alpha _n, \end{aligned}$$

where

$$\begin{aligned} H_n (A) = (-1)^n \exp (A^2) \frac{d^n}{dA^n} \exp (-A^2), \quad \alpha _n = (2^{n-1} n ! \sqrt{\pi })^{1/2} \end{aligned}$$

$\alpha _n$ being taken in such a way that for all $n \in \mathbb {N}$

$$\begin{aligned} \int _0^{+\infty } \psi _n(A)^2 dA= 1. \end{aligned}$$

Such eigenfunctions satisfy the properties

$$\begin{aligned} \sqrt{2n} \psi _{n-1}(A)= & {} A \psi _n(A) + \psi _n'(A), \\ \psi _n'(A)= & {} \sqrt{\frac{n}{2}} \psi _{n-1}(A) -\sqrt{\frac{n+1}{2}} \psi _{n+1}(A), \\ A\psi _{n}(A)= & {} \sqrt{\frac{n}{2}} \psi _{n-1}(A)+ \sqrt{\frac{n+1}{2}}\psi _{n+1}(A), \end{aligned}$$

so that

$$\begin{aligned} A \psi _n' = n \psi _n -A^2 \psi _n + \sqrt{n(n-1)} \psi _{n-2}, \end{aligned}$$

and

$$\begin{aligned} (A \psi _n(A))' = (n+1) \psi _n -A^2 \psi _n + \sqrt{n(n-1)} \psi _{n-2}. \end{aligned}$$

Because we restrict to $A \in (0,+\infty )$, we only consider the case of even integers $n = 2k$, $k \in {\mathbb {N}}$. As a matter of fact, $\psi _{2k}$ (resp. $\psi _{2k+1}$) are even functions (resp. odd functions): in order to comply with homogeneous Neumann boundary conditions on $A=0$, *n* needs to be even. Let us also observe that

$$\begin{aligned} \int _0^{+\infty } \psi _{2k} (A) dA = {{\tilde{\alpha }}}_k , \quad \hbox {where} \quad {{\tilde{\alpha }}}_k = \left( \frac{ (2k) ! \sqrt{\pi } }{2^{2k} (k!)^2 } \right) ^{1/2} \quad \hbox {for all} \quad k\ge 1. \end{aligned}$$

Then, in order to represent solutions of (11) we decompose *S*(*t*, *a*) as a finite sum of $(\psi _{2k})_{k \in \mathbb {N}})$ eigenfunctions:

$$\begin{aligned} S(t,a) = \sum _{k=0}^K {\overline{S}}_k(t) \psi _{2k}\left( \frac{a}{\uplambda (t)} \right) \frac{1}{\uplambda (t)}. \end{aligned}$$

We provide detailed computations below (where $A = a / \uplambda$)

$$\begin{aligned}{} & {} \partial _t S(t,a) = \sum _{k=0}^K \left( \frac{{{\dot{{\overline{S}}}}}_k }{\uplambda } - \frac{{\overline{S}}_k {{\dot{\uplambda }}}}{\uplambda ^2} \right) \psi _{2k}(A) - \frac{{\overline{S}}_k {{\dot{\uplambda }}}}{\uplambda ^2} A \psi _{2k}'(A), \\{} & {} - \left( \beta _0 + a^2 \beta _1 \right) I S = - \sum _{k=0}^K \left( \beta _0 + \uplambda ^2 A^2 \beta _1 \right) \frac{I {\overline{S}}_k}{\uplambda } \psi _{2k}(A), \\{} & {} - \partial _a (\mu _b S) = \frac{\mu _0}{\uplambda }\sum _{k=0}^K \partial _A \left( A \psi _{2k}(A) \right) {\overline{S}}_k, \\{} & {} D \partial _a^2 S = \frac{D}{\uplambda ^3} \sum _{k=0}^K {\overline{S}}_n \psi ''_{2k}(A). \end{aligned}$$

Therefore, one has

$$\begin{aligned}{} & {} \sum _{k=0}^K \left( \frac{{{\dot{{\overline{S}}}}}_k }{\uplambda } - \frac{{\overline{S}}_k {{\dot{\uplambda }}} }{\uplambda ^2} - 2k \frac{{\overline{S}}_k {{\dot{\uplambda }}}}{\uplambda ^2}\right) \psi _{2k}(A) + \sum _{k=0}^K \frac{{\overline{S}}_k {{\dot{\uplambda }}}}{\uplambda ^2} A^2 \psi _{2k}(A) \\{} & {} \qquad - \sum _{k=0}^K \sqrt{2k(2k-1)} \frac{{\overline{S}}_k {{\dot{\uplambda }}}}{\uplambda ^2} \psi _{2k-2}(A) \\{} & {} \quad = \sum _{k=0}^K \left( - \beta _0 \frac{I {\overline{S}}_k}{\uplambda } + (2k+1) \frac{\mu _0 {\overline{S}}_k}{\uplambda }- (4k +1 ) \frac{D {\overline{S}}_k}{\uplambda ^3} \right) \psi _{2k} (A) \\{} & {} \qquad + \sum _{k=0}^K \left( - \beta _1 \uplambda ^2 \frac{I {\overline{S}}_k}{\uplambda } + \frac{D {\overline{S}}_k}{\uplambda ^3} - \frac{\mu _0 {\overline{S}}_k}{\uplambda }\right) A^2 \psi _{2k}(A) \\{} & {} \qquad + \sum _{k=0}^K \sqrt{2k(2k-1)} \frac{\mu _0 {\overline{S}}_k }{\uplambda } \psi _{2k-2}(A). \end{aligned}$$

Identifying for each $k \in \{ 1 \dots K \}$ the coefficients in front of $A^2 \psi _{2k} (A)$ then gives

$$\begin{aligned} \frac{ {{\dot{\uplambda }}}}{\uplambda } = -\beta _1 I \uplambda ^2 - \mu _0 + \frac{D }{\uplambda ^2} . \end{aligned}$$

On the other hand, the dynamics of the *K* coefficients is given by

$$\begin{aligned}{} & {} \frac{{{\dot{{\overline{S}}}}}_k }{\uplambda } - (2k+1) \frac{{\overline{S}}_k {{\dot{\uplambda }}}}{\uplambda ^2} - \sqrt{2(k+1)(2k+1)} \frac{{\overline{S}}_{k+1} {{\dot{\uplambda }}}}{\uplambda ^2} \\{} & {} \quad = - \beta _0 \frac{I {\overline{S}}_k}{\uplambda } + (2k+1) \frac{\mu _0 {\overline{S}}_k}{\uplambda }- (4k +1 ) \frac{D {\overline{S}}_k}{\uplambda ^3} + \sqrt{2(k+1)(2k+1)} \frac{\mu _0 {\overline{S}}_{k+1}}{\uplambda }, \end{aligned}$$

for $k = 0, \dots , K-1$ and for $k=K$

$$\begin{aligned}{} & {} \frac{{{\dot{{\overline{S}}}}}_K }{\uplambda } - (2K+1) \frac{{\overline{S}}_K {{\dot{\uplambda }}}}{\uplambda ^2} \\{} & {} \quad = - \beta _0 \frac{I {\overline{S}}_K}{\uplambda } + (2K+1) \frac{\mu _0 {\overline{S}}_K}{\uplambda }- (4K +1 ) \frac{D {\overline{S}}_k}{\uplambda ^3} . \end{aligned}$$

Hence for $$k=0, \dots , K-1$$

$$\begin{aligned} {{\dot{{\overline{S}}}}}_k= & {} - \beta _0 I {\overline{S}}_k - (2k+1) \beta _1 \uplambda ^2 I {\overline{S}}_k -2k\frac{D {\overline{S}}_k }{\uplambda ^2} \\{} & {} \quad + \sqrt{2(k+1)(2k+1)} \, {\overline{S}}_{k+1}\left( \frac{D }{\uplambda ^2} -\beta _1 I \uplambda ^2 \right) , \end{aligned}$$

and for $$k=K$$

34 $$\begin{aligned} {{\dot{{\overline{S}}}}}_K = - \beta _0 I {\overline{S}}_K - (2K+1) \beta _1 \uplambda ^2 I {\overline{S}}_K -2K\frac{D {\overline{S}}_K }{\uplambda ^2}. \end{aligned}$$

In other words, one has

35 $$\begin{aligned} \frac{d \textbf{S}}{dt} = - \left( \beta _0 + \beta _1 \uplambda ^2 \right) I \, \textbf{S} + \left( \frac{D}{\uplambda ^2} -\beta _1 I \uplambda ^2 \right) \textbf{G}_1 \cdot \textbf{S} - \left( 2 \beta _1 I \uplambda ^2 + \frac{2D}{\uplambda ^2} \right) \textbf{G}_0 \cdot \textbf{S} \end{aligned}$$

where

$$\begin{aligned} \textbf{S}= & {} \begin{bmatrix} {\overline{S}}_0 \\ \vdots \\ {\overline{S}}_K \end{bmatrix}, \ \ \textbf{G}_0 = \hbox {Diag} \left( 0, 1, \dots , K-1, K \right) , \\ \textbf{G}_1= & {} \begin{bmatrix} 0 &{} \nu _0 &{} &{} &{} \\ &{} \ddots &{} \ddots &{} &{} \\ &{} &{} \ddots &{} \nu _{K-2} &{} \\ &{} &{} &{} \ddots &{} \nu _{K-1}\\ &{} &{} &{} &{} 0 \end{bmatrix} \quad \hbox {where} \quad \nu _k = \sqrt{2(k+1)(2k+1)}. \end{aligned}$$

The dynamics of infected follows from the computation

$$\begin{aligned} \int _0^{+\infty } \left( \beta _0 + \beta _1 \uplambda ^2 A^2 \right) \psi _{2k}(A)\, dA = {{\tilde{\alpha }}}_k \left( \beta _0 + \uplambda ^2 (4 k +1 ) \beta _1\right) , \end{aligned}$$

so that

$$\begin{aligned} \frac{dI}{dt} = {\overline{\beta }}I\sum _{k=0}^K {{\tilde{\alpha }}}_k {\overline{S}}_k - \gamma I. \end{aligned}$$

where

36 $$\begin{aligned} {\overline{\beta }}= \beta _0 + \beta _1 \uplambda ^2 \left( 1 + 4\frac{\sum _{k=0}^K k {{\tilde{\alpha }}}_k {\overline{S}}_k}{ \sum _{k=0}^K {{\tilde{\alpha }}}_k {\overline{S}}_k}\right) . \end{aligned}$$

Note that contrary to the case $K=0$, it is more convenient to keep $\uplambda$ as a variable rather than ${\overline{\beta }}$. To summarize, denoting

$$\begin{aligned} {{\mathcal {S}}} = \sum _{k=0}^K {{\tilde{\alpha }}}_k {\overline{S}}_k, \end{aligned}$$

the generalized model expresses as

37 $$\begin{aligned} \left\{ \begin{aligned}&\frac{d {{\mathcal {S}}}}{dt} = - {\overline{\beta }}I {{\mathcal {S}}}, \\&\frac{d I}{dt} = I \left( {\overline{\beta }}{{\mathcal {S}}} - \gamma \right) , \\&\frac{d R}{dt} = \gamma I, \\&\frac{ d\uplambda }{dt} = -\beta _1 I \uplambda ^3 - \mu _0 \uplambda + \frac{D }{\uplambda }, \end{aligned} \right. \end{aligned}$$

where ${\overline{\beta }}$ is defined by (36) and the dynamics of $( {\overline{S}}_k )_{k=0\dots K}$ is given by (35). The initial conditions read as:

38 $$\begin{aligned} \left\{ \begin{aligned}&\uplambda (0)=1, \\&{\overline{S}}_k(0) = {\overline{S}}_{k,0}, \quad k=0\dots K\\&I(0)=I_0 \ge 0, \end{aligned} \right. \end{aligned}$$

In such a way that

$$\begin{aligned} I_0 + \sum _{k=0, \dots ,K} {{\tilde{\alpha }}}_k {\overline{S}}_{k,0} =1. \end{aligned}$$

We claim that

#### Theorem 5

Let $({\overline{S}}_k)_{k=0 \dots K}$, *I* and *R* be solution of (37), (36), (35) with initial conditions (38). Assume that $\mu _0>0$ and $D>0$. Then, as time *t* goes to $+\infty$,

$$\begin{aligned} \uplambda (t) \rightarrow \uplambda ^\infty = \sqrt{\frac{D}{\mu _0}}>0, \quad {\overline{S}}_0(t) \rightarrow {\overline{S}}_0^\infty >0, \quad {\overline{S}}_k(t) \rightarrow 0, \quad k\ge 1, \\ I(t) \rightarrow 0, \quad {{\mathcal {S}}}(t) \rightarrow {{\mathcal {S}}}^\infty = {{\tilde{\alpha }}}_0 {\overline{S}}_0^\infty \in (0,N), \quad R(t) \rightarrow R^\infty \in (0,N) , \end{aligned}$$

where $${{\mathcal {S}}}^\infty +R^\infty =N$$.

#### Proof

Since most of the arguments are similar to the case $K=1$, we only sketch the proof. First the convergence of ${{\mathcal {S}}}$, *I* and *R* to limit values ${{\mathcal {S}}}^\infty$, 0 and $R^\infty$ follows from the same arguments. Next, the convergence of $\uplambda (t)$ to $\uplambda ^\infty$ is also identical to the case $K=1$. Then, the convergence of ${\overline{S}}_K(t)$ to 0 follows from (34). The case $k = 0\dots K-1$ follows from the following lemma:

#### Lemma 1

Let $t\mapsto x(t)$ be defined over $\mathbb {R}^+$ satisfy

$$\begin{aligned} \dot{x} (t)= - c(t) x(t) + b(t), \end{aligned}$$

for some $c(t)>0$ and *b*(*t*) such that $(c(t),b(t)) \rightarrow (c^\infty , b^\infty )$ with $c^\infty >0$. Then $x(t) \rightarrow 0$ as *t* goes to $+\infty$.

*Sketch of proof*: replacing *x* by $x - b^\infty / c^\infty$, one may assume that $b^\infty =0$. Then,

$$\begin{aligned} x(t) = \exp \left( - \int _0^t c(s)ds\right) x_0 + \int _0^t \exp \left( - \int _s^t c (\tau ) d\tau \right) b(s) ds. \end{aligned}$$

The first term in the above right hand side converges to zero because of asymptotic properties of *c*(*t*), and the second one can be estimated for instance by splitting the integral over (0, *T*) and (*T*, *t*) for $t \ge T>0$. $\square$

Let us finally observe that the limit transmission rate satisfies

$$\begin{aligned} {\overline{\beta }}_\infty = \beta _0 + \beta _1 \frac{D}{\mu _0}, \end{aligned}$$

which is the same as the average limit transmission rate ${\overline{\beta }}_\infty$ associated with solutions of the partial differential equation model.

### Numerical simulations

We make comparisons between solutions of the full partial differential equations model and solutions obtained from Ordinary Differential Equations of order K as derived in the previous Sect. 6.1. The initial profile of susceptible individuals in terms of trait variable $a \in {\mathbb {R}}^+$ is taken as follows:

$$\begin{aligned} S_0(a) = {\overline{S}}_0 a \exp (-a), \quad \hbox {with} \quad {\overline{S}}_0 = 1 - I_0 \end{aligned}$$

and initial rate of infected individuals $I_0=3\times 10^{-5}$ (considering $N=1$). The transmission rate model parameters are

$$\begin{aligned} \beta _0= 0.01 \, \hbox {days}^{-1}, \qquad \beta _1= 0.18 \, \hbox {days}^{-1} \end{aligned}$$

**Fig. 3:**
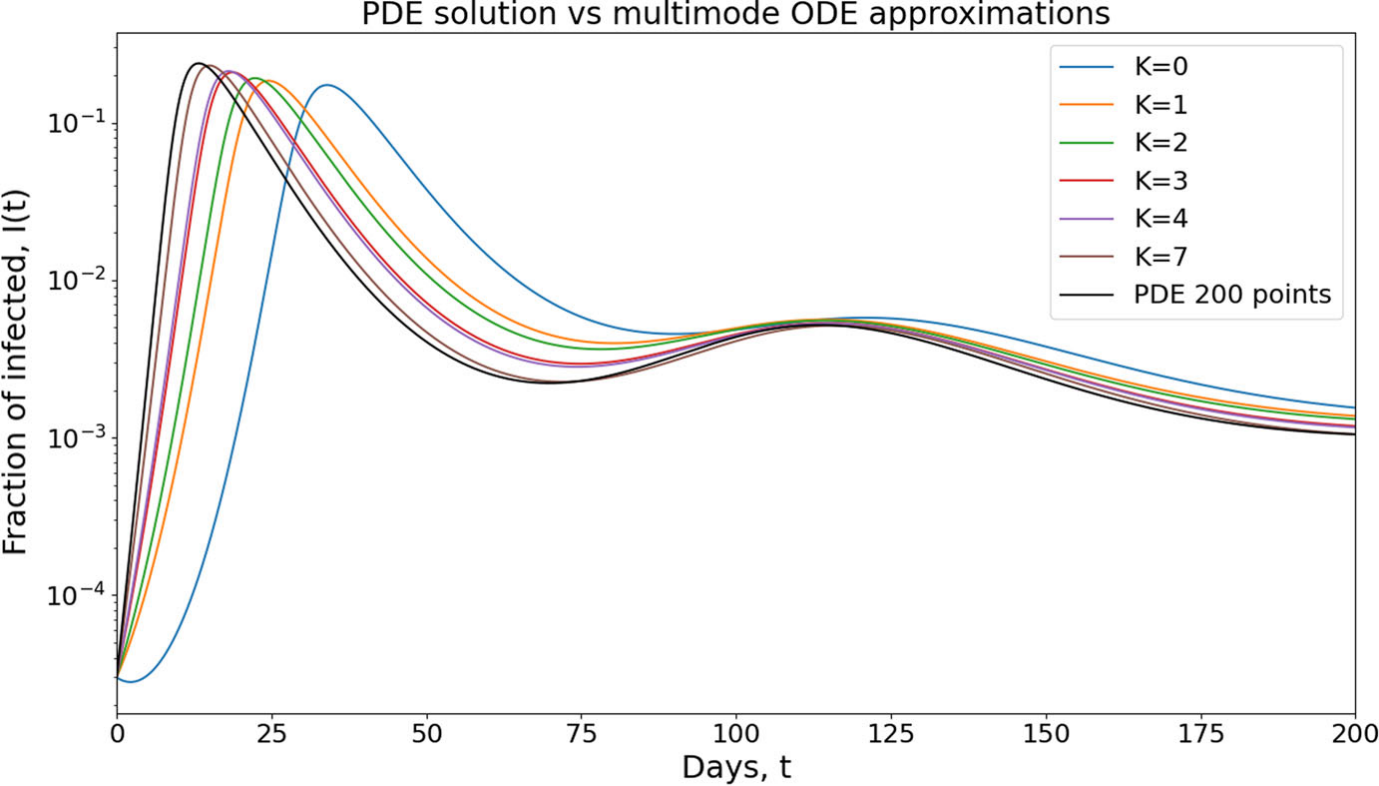
Rate of infected individuals *I*(*t*)/*N* solution of ODE for several orders of approximations $K=0,1,2,3,4$ and 7, and PDE solution (black curve)

Drift and diffusion coefficients are equal to

$$\begin{aligned} \mu _0= 5\times 10^{-3} \, \hbox {days}^{-1}, \quad D= 0.08 \, \hbox {days}^{-1}, \end{aligned}$$

and $\gamma = 0.25\, \hbox {days}^{-1}$, i.e. ${{\mathcal {R}}} = 2.89$. The simulation is performed over time interval [0, 200] (in days) for both PDE or ODE approaches. Space discretization indicates that 200 points in space are enough to achieve reasonable convergence of solutions of the full PDE system. Simulations of the ODE system associated with $K=0,1,2,3,4,7$ are illustrated in Fig. 3 above.^2^

We observe a somehow monotonous convergence behavior when *K* becomes large enough, in particular for the maximum level of *I*(*t*) and time for such maximum and the period of oscillations. Further investigations will be needed to assess the epidemiological relevance of such higher order approximation models, which allows us to consider general initial probability densities $f_0$ beyond truncated Gaussian distributions.

## Discussion

### Main results

We have derived here a system describing the evolution of epidemics taking into account the behavioral heterogeneity and variability of the population of susceptibles. Individual behaviors are characterized by a compound risk variable $a \in {\mathbb {R}}^+$ associated with the susceptibility with respect to the epidemic. Larger values of *a* correspond to increasing exposure to contagion, whereas low values are associated with little exposed behaviors. Thus, the population of susceptibles is structured by this variable *a*: $S=S(t,a)$, and the classical transmission coefficient $\beta =\beta (a)$ is an increasing function of *a*. Rather than working with *S*(*t*, *a*), we found it more revealing to write the equations in terms of the total population ${\overline{S}}(t)= \int _0^\infty S(t,a) da$ and the probability distribution of the trait *a* in the population: $f(t,a)= S(t,a)/{\overline{S}}(t)$.

We first expressed the effect of the epidemics on the distribution *f*(*t*, *a*) as a transport equation. We also assumed that this distribution is subject to random fluctuations according to a drift-diffusion stochastic process. Combining these two effects, we derived a Fokker–Planck equation governing the evolution of the distribution *f*. This equation is coupled with the evolution equations for ${\overline{S}}(t)$ and *I*(*t*), the total numbers of infected. We then derived a system, that we call the *SfIR* system, standing for the variables ${\overline{S}}(t)$, *f*(*t*, *a*), *I*(*t*) and the population of recovered *R*(*t*). This new system is non-linear and non-local and combines a partial differential equation of Fokker–Planck type and ordinary differential equations for ${\overline{S}}$ and *I*. The *SfIR* model is a natural extension of the classical *SIR* compartmental model to populations when considering heterogeneity and variability of individual behaviors.

Numerical simulations of this system showed that it exhibits some of the trademark dynamics of the unfolding of epidemics such as the current COVID-19 epidemic. Namely, we observed plateaus, shoulders, oscillations and rebounds. These aspects do not emerge in the classical *SIR* model and can be viewed as a consequence of including individual behaviors, heterogeneity, and variability.

We then studied the question of large time behavior for this system. There is a long-standing literature devoted to this question in the case of the scalar Fokker–Planck equation by itself which turns out to be rather delicate. This new context of a coupled non-linear and non-local system further complicates matters and requires new developments on this question. Under some assumptions on the model coefficients, i.e. transmission rate, drift and diffusion, we were able to prove the convergence of the probability distribution $f(t, \cdot )$ to a unique equilibrium distribution $f_\infty$. This result extends the classical convergence to equilibrium results for solutions to Fokker–Planck equations in the absence of epidemic coupling, under classical Bakry–Emery (Bakry and Émery 1985) hyper-contractivity assumptions on drift and diffusion coefficients.

We paid particular attention to the case of constant diffusion *D*, linear dependence of the drift with respect to *a* and affine dependence of the transmission rate with respect to $a^2$. We showed that this framework is associated with an Ornstein-Uhlenbeck process for the fluctuations of *a*. In this case, we further derived a model of reduced complexity. This ODE system was obtained by looking for self-similar solutions of the equation governing the distribution *f* involving Gaussian probability distributions truncated over ${\mathbb {R}}^+$. We showed that this ODE system even though quite simple still captures epidemic patterns such as rebounds, shoulders, and plateaus.

We also obtained higher order ODE models approximating the solutions of the PDE system through spectral type methods involving the eigenfunctions of some associated operator akin to the quantum harmonic oscillator. Furthermore, we illustrated the convergence to the PDE solution in the presence of oscillations as the model order increases.

Finally, we stated and proved some mathematical properties of the reduced complexity ODE system such as large time behavior depending on various assumptions on the model coefficients.

### Perspectives

The derivation of the *SfIR* model, and our first analytical results naturally open many perspectives, both in the epidemiological modeling and in the mathematical analysis of these systems. The present paper is a first stage of a research program on epidemiological modeling in this vein.

The new system we introduced here raises a number of open mathematical questions. To start with, the existence and uniqueness of the solution to the Cauchy problem associated with this system is still open. As already noted, this system combines the difficulties of being non-linear and non-local. The extension of the convergence to equilibrium result by relaxing the assumptions and allowing as general a framework as possible is a natural question. In particular, deriving regularity and integrability of solutions for a wide class of initial data is open. It would be interesting to further describe the total number of infected individuals after the epidemic has subsided and how this number depends on the various parameters. More generally, it would be desirable to better understand the qualitative properties of dependence of solutions and their final states with respect to the parameters. Further mathematical developments are needed to rigorously justify this reasoning and to explore the qualitative basis for and quantitative levels of oscillations.

From an epidemiological point of view, one is naturally led to investigate extensions of the approach developed here. First, our model here rested on the assumption of *homogeneous mixing*. It would be interesting to expand our approach to the more complex and more realistic framework of *preferential mixing* (Feng 2014). Other natural extensions of the *SIR* model take into account additional epidemiological phenomena, e.g., loss of immunity and subsequent reinfection (Lavine et al. 2021) and the inclusion of an explicit exposed period before contagion as in *SEIR* models or via the use of realistic generation interval distributions (Park et al. 2022).

Second, as observed in the current COVID-19 pandemic, the succession of variants plays a major role in the unfolding of epidemics. It is therefore natural to use the *SfIR* methodology in the presence of variants. Such an approach could be used to model competition phenomenon between variants characterized by different parameters regarding transmission rate $\beta$ and average infection duration $\gamma ^{-1}$. In all these frameworks, it is natural to seek models of reduced complexity.

Third, the recent COVID-19 pandemic also showed the importance of “awareness” and “fatigue” phenomena, which were modeled in Weitz et al. (2020a) via a top-down approach. In the spirit of that work, embedding state- and/or time-dependence in the PDE framework or in the ODE model parameters is likely to enrich the capacity of *SfIR* modeling approach to capture more complex global phenomena. Specifically, we note that in the *SfIR* PDE framework, awareness-driven changes in behavior can dynamically shift susceptibility distributions in a way that is different than contagion-driven sculpting—evaluating the combination of both sculpting and shifting could lead to productive avenues for both theory and model-data integration.

Finally, combining the *SfIR* modeling approach with spatial diffusion of infection is undoubtedly a challenging but very relevant issue to address in the future. One may contemplate combining the models we have introduced here for a single location to include spatial diffusion (Roques et al. 2020) or coupling between locations that may differ in their response policies (Kortessis et al. 2020). In doing so, recent measurement campaigns of SARS-CoV-2 in wastewater in the framework of surveillance networks throughout the world is a motivation to extend our modeling approach to Wastewater Based Epidemiology—as a means to link ongoing changes in local incidence to forecasting and mitigation.

## Appendix: Parsimonious model with more general probability distribution profiles

Section 4 focused on Gaussian distribution profiles. We consider here more general profiles of susceptible individuals for which self similar solutions exist.

### Assumptions

We make the following assumptions:

- The initial distribution $f_0$ of susceptible population is described in terms of the continuous risk variable $a \in \mathbb {R^+}$
  $$\begin{aligned} S_0(a) = {\overline{S}}_0 f_0 (a) \ \ \hbox {with} \ \ f_0(a) = \frac{1}{\uplambda _0} \phi (\frac{a}{\uplambda _0}), \end{aligned}$$
  where for some given real $k>0$
  39 $$\begin{aligned} \phi (a) = k^{-1/k}\exp \left( - \frac{a^k}{k}\right) / \Gamma \left( \frac{1}{k} +1 \right) , \end{aligned}$$
  where $\Gamma$ denotes the classical Gamma function
  $$\begin{aligned} \Gamma (z) = \int _0^{+\infty } x^{z-1} \exp (-x) dx. \end{aligned}$$
- Diffusion coefficient depending on *a* and *t*
  $$\begin{aligned} \frac{\sigma ^2 }{2} = D(t) a^{2-k} \quad \hbox {for some positive function} \quad t \mapsto D(t). \end{aligned}$$
- The transmission rate function $\beta$ is a power function of *a*
  40 $$\begin{aligned} \beta (a) = \beta _0 + \beta _1 a^k. \end{aligned}$$
- Background drift effect
  $$\begin{aligned} \mu _b (t,a) = - \mu _0(t) a^{k-1} + D(t) (k-2) a^{2-k} . \end{aligned}$$
  Note again that $\mu _0(t)$ may be any non-negative function of time only, and that (unless $k=2$) an extra diffusion dependent drift is also needed.
- The susceptible population *S*(*t*, *a*) is assumed to be expressed as a self-similar profile
  41 $$\begin{aligned} S(t,a) = \frac{{\overline{S}}(t)}{\uplambda (t)} \phi \left( \frac{a}{\uplambda (t)} \right) , \end{aligned}$$
  for some functions of time $t \mapsto {\overline{S}}(t)$ and $t \mapsto \uplambda (t)$ such that ${\overline{S}}(0)={\overline{S}}_0$ and $\uplambda (0)=\uplambda _0$.

Let us observe at this stage that

$$\begin{aligned} \phi '(A) = -A^{k-1} \phi (A). \end{aligned}$$

### Derivation

We deduce from the first equation of (11) that

$$\begin{aligned}{} & {} \left( \frac{{\dot{{\overline{S}}}}}{\uplambda } - \frac{{{\dot{\uplambda }}} {\overline{S}}}{\uplambda ^2} \right) \phi (A) - A \phi '(A) \frac{{{\dot{\uplambda }}} {\overline{S}}}{\uplambda ^2} = - \frac{I \left( \beta _0 + \beta _1\uplambda ^k A^k \right) {\overline{S}}}{N \uplambda } \phi (A) \\{} & {} \quad +\, \frac{\mu _0 {\overline{S}}}{ \uplambda } \frac{d\left[ A \phi (A) \right] }{dA} + \frac{D {\overline{S}}(k-2)}{\uplambda ^{k+1}} \frac{d \left[ A^{1-k} \phi (A) \right] }{dA} + \frac{D {\overline{S}}}{ \uplambda ^{k+1}} \frac{d^2\left[ A^{2-k} \phi (A) \right] }{dA^2} . \end{aligned}$$

Observing that

$$\begin{aligned}{} & {} \frac{d\left[ A \phi (A) \right] }{dA} = \phi (A) - A^k \phi (A), \\{} & {} \frac{d \left[ A^{1-k} \phi (A) \right] }{dA} = (1-k)A^{-k} \phi (A) - \phi (A), \end{aligned}$$

and

$$\begin{aligned} \frac{d^2\left[ A^{2-k} \phi (A) \right] }{dA^2} = (2-k)(1-k) A^{-k} \phi (A) + (k-3) \phi (A) + A^k \phi (A), \end{aligned}$$

one concludes that

42 $$\begin{aligned}{} & {} \phi (A) \left[ \left( \frac{{\dot{{\overline{S}}}}}{\uplambda } - \frac{{{\dot{\uplambda }}} {\overline{S}}}{\uplambda ^2} \right) + \frac{I \beta _0 {\overline{S}}}{N \uplambda } - \frac{\mu _0 {\overline{S}}}{ \uplambda } + \frac{D {\overline{S}}}{\uplambda ^{k+1} } \right] \nonumber \\{} & {} \quad = A^k \phi (A) \left[ - \frac{{{\dot{\uplambda }}} {\overline{S}}}{\uplambda ^2} - \frac{I \beta _1\uplambda ^k {\overline{S}}}{N \uplambda } - \frac{\mu _0 {\overline{S}}}{\uplambda } + \frac{D {\overline{S}}}{\uplambda ^{k+1} } \right] \end{aligned}$$

We therefore require that the two time dependent functions on the left and right hand side of (27) are equal to zero:

43 $$\begin{aligned}{} & {} \frac{{{\dot{\uplambda }}}}{\uplambda } = - \frac{I \beta _1\uplambda ^k}{N } - \mu _0 + \frac{D}{\uplambda ^k } \end{aligned}$$

44 $$\begin{aligned}{} & {} \left( \frac{{\dot{{\overline{S}}}}}{\uplambda } - \frac{{{\dot{\uplambda }}} {\overline{S}}}{\uplambda ^2} \right) + \frac{I \beta _0 {\overline{S}}}{N \uplambda } - \frac{\mu _0 {\overline{S}}}{\uplambda } + \frac{D {\overline{S}}}{\uplambda ^{k+1} } = 0 \end{aligned}$$

hence substituting (27) into (27), one gets on the one hand

45 $$\begin{aligned} \frac{{\dot{{\overline{S}}}}}{{\overline{S}}} = - \frac{I \left( \beta _0 + \beta _1 \uplambda ^k\right) }{N}. \end{aligned}$$

On the other hand, the second equation of (11) leads to

46 $$\begin{aligned} \frac{dI}{dt} = I \left( \frac{{\overline{S}}( \beta _0 + \beta _1 \uplambda ^k )}{N} - \gamma \right) \end{aligned}$$

As a consequence, the derived model writes as follows

47 $$\begin{aligned} \boxed { \left\{ \begin{aligned} \displaystyle \frac{d{{\overline{S}}}}{dt }&= \displaystyle - \frac{{\overline{S}}I \left( \beta _0 + \beta _1 \uplambda ^k\right) }{N} \\ \displaystyle \frac{d \uplambda }{dt}&= \displaystyle - \frac{I \beta _1\uplambda ^{k+1}}{N} - \mu _0 \uplambda + \frac{D}{\uplambda } \\ \displaystyle \frac{d I}{dt}&= \displaystyle \frac{{\overline{S}}I ( \beta _0 + \beta _1 \uplambda ^k )}{N} - \gamma I \\ \displaystyle \frac{dR}{dt}&= \gamma I, \end{aligned} \right. } \end{aligned}$$

with initial conditions ${\overline{S}}(0) = {\overline{S}}_0$, $\uplambda (0)=\uplambda _0$, $I(0)= I_0$ and $R(0)=0$.

### Dynamics of the average transmission rate ${\overline{\beta }}$

Le us recall the equation governing the probability density function $f(t,a)=S(t,a)/{\overline{S}}(t)$ of susceptible individuals

$$\begin{aligned} \partial _t f(t,a) = - \frac{I(t)}{N} f(t,a) \left( \beta (a) - {\overline{\beta }}(t) \right) - \partial _a (\mu _b(t,a) f(t,a)) + \partial _{aa}^2 \left( D a^{2-k} f(t,a) \right) . \end{aligned}$$

Then, we introduce the notation

$$\begin{aligned} {\overline{\psi }} (t) = \int _{{\mathbb {R}}^+} \psi (a) f(t,a) \, da, \end{aligned}$$

so that one has

$$\begin{aligned}{} & {} {\overline{\beta }}(t) = \int _{{\mathbb {R}}^+} \beta (a) f(t,a) \, da \\{} & {} Var(\beta )(t) = \overline{\beta ^2}(t) - {\overline{\beta }}(t)^2. \end{aligned}$$

Then, inserting the self similar profiles into (4.3), $f(t,a) = \uplambda (t)^{-1} \phi (a/\uplambda (t))$, so that one gets

$$\begin{aligned}{} & {} {\overline{\beta }}(t) = \beta _0 + \beta _1 \uplambda ^k. \\{} & {} \overline{\beta ^2}(t) = \int _{{\mathbb {R}}^+} (\beta _0^2 + 2 \beta _0 \beta _1 \uplambda ^k A^k + \uplambda ^{2k} \beta _1^2 A^{2k}) \phi (A) dA = \beta _0^2 + 2 \beta _0 \beta _1 \uplambda ^k + (k+1) \beta _1^2 \uplambda ^{2k}. \end{aligned}$$

The variance $Var(\beta )(t)$ can then be expressed as

$$\begin{aligned} Var(\beta )(t) = k \beta _1^2 \uplambda ^{2k}(t) = k ({\overline{\beta }}- \beta _0)^2 \end{aligned}$$

It means that the dynamics of $t \mapsto \uplambda (t)$ is directly linked to the average value of the $\beta$ parameter. Therefore, the equation $\uplambda$ can be replaced by the dynamics of the average transmission rate ${\overline{\beta }}$ in System (27)

$$\begin{aligned} \frac{d {\overline{\beta }}}{dt} =- k \frac{I}{N} ({\overline{\beta }}- \beta _0)^2 - k \mu _0 ({\overline{\beta }}- \beta _0) + k D \beta _1 \uplambda ^{k-2}. \end{aligned}$$

Therefore, (27) becomes

48 $$\begin{aligned} \boxed { \left\{ \begin{aligned} \displaystyle \frac{d{{\overline{S}}}}{dt }&= \displaystyle - \frac{{\overline{\beta }}\, {\overline{S}}\, I }{N} \\ \displaystyle \frac{d {\overline{\beta }}}{dt}&= \displaystyle - k \frac{I}{N} ({\overline{\beta }}- \beta _0)^2 - k \mu _0 ({\overline{\beta }}- \beta _0) + k D \beta _1^{3-k} ({\overline{\beta }}- \beta _0)^{k-2}\\ \displaystyle \frac{d I}{dt}&= \displaystyle \frac{ {\overline{\beta }}\, {\overline{S}}\, I}{N} - \gamma I \\ \displaystyle \frac{dR}{dt}&= \gamma I, \end{aligned} \right. } \end{aligned}$$

with initial conditions $I(0) = I_0 \in (0,N)$, ${\overline{S}}(0) = N-I_0$, $R(0)=0$ and ${\overline{\beta }}(0) = {\overline{\beta }}_0 \ge \beta _0$.

## Appendix: Csiszár–Kullback–Pinsker inequality

One of the key estimate in the proof of convergence to equilibrium of the distribution *f* in Theorems 2 and 4 is the estimate of the $L^1({\mathbb {R}}^+)$ distance of two probability distributions in terms of the associated relative entropy. This is known as Csiszár–Kullback–Pinsker’s inequality:

### Theorem 6

(Csiszár–Kullback–Pinsker) Let *f*, *g* be two non-negative real functions in $L^1({\mathbb {R}}^+)$ with $\Vert f\Vert _1=\Vert g\Vert _1 = 1$. It holds that

49 $$\begin{aligned} \left\| f - g \right\| _1^2 \le 2 \int _{{\mathbb {R}}^+} f \log \frac{f}{g}. \end{aligned}$$

For the readers convenience, we provide here a simple and elegant proof due to J.A. Canizo (https://canizo.org/page/28).

### Proof

A preliminary step is to observe that for all $r \ge 0$, one has:

$$\begin{aligned} \Phi (r):= r \log r - r + 1 \ge \frac{3}{2} \frac{(r-1)^2}{r+2}. \end{aligned}$$

Then, such estimate combined by Cauchy-Schwarz inequality leads

$$\begin{aligned} \Vert f - g \Vert _1^2 = \left( \int _{{\mathbb {R}}^+} \left| \frac{f}{g} - 1 \right| g \right) ^2 \le \int _{{\mathbb {R}}^+ } \frac{ \displaystyle \left( \frac{f}{g} - 1 \right) ^2}{\displaystyle \frac{f}{g} + 2 } g \int _{{\mathbb {R}}^+ } \left( \frac{f}{g} + 2 \right) g, \\ \qquad \qquad = 3 \int _{{\mathbb {R}}^+ } \frac{ \displaystyle \left( \frac{f}{g} - 1 \right) ^2}{\displaystyle \frac{f}{g} + 2 } g \le 2 \int _{{\mathbb {R}}^+} \Phi \left( \frac{f}{g} \right) g = 2 \int _{{\mathbb {R}}^+} f \log \frac{f}{g} . \end{aligned}$$

$$\square $$

## Appendix: Decay at infinity of solutions of the SfIR system

We consider the System (12) and focus here on the case when $\mu _b$ and *D* do not depend on time *t*. The aim of this appendix is to provide some decay properties of solutions *f* of System (12) which allows us to justify multiple integration by parts and other formal computations we carried in Sect. 3, at least in the Gaussian like case. We leave the general case open.

Assuming that conditions (*H*2) and (*H*4) hold, that is that diffusion is non-degenerate and the probability density $f_\infty$ decreases at infinity at least as a Gaussian distribution. In this appendix, in addition to the assumptions of Sect. 3, we suppose that the following conditions hold. First, the diffusion *D* is bounded:

$$\begin{aligned} D(a) \le D^* < + \infty \quad \hbox {for all} \quad a \in {\mathbb {R}}^+. \end{aligned}$$

The transmission rate $\beta$ is growing at most linearly in $a^2$:

$$\begin{aligned} \beta (a) \le C (1+a^2) \quad \hbox {for all} \quad a \in {\mathbb {R}}^+ \end{aligned}$$

(where here and in the following *C* denotes a generic positive constant). Lastly, the equilibrium $f_\infty$ satisfies:

$$\begin{aligned} \left| \frac{f_\infty '(a)}{f_\infty (a)} \right| = \left| \alpha '(a) \right| \le C(1+a) \quad \hbox {for all} \quad a \in {\mathbb {R}}^+. \end{aligned}$$

In this framework, we have the following a priori estimate.

### Theorem 7

Assume that (*H*2), (*H*4) and the previous assumptions on $D, \beta , f_\infty$ hold. Furthermore, suppose that the initial profile of susceptibles $S_0$ satisfies

$$\begin{aligned} S_0 \in L^1({\mathbb {R}}^+), \ \ \int _{{\mathbb {R}}^+} S_0(a)\, da \le 1, \end{aligned}$$

and

$$\begin{aligned} 0 \le S_0(a) \le M f_\infty (a) < + \infty \ \ \hbox {for a.e.} \ \ a \in {\mathbb {R}}^+ \end{aligned}$$

for some positive constant $M>0$. Then, the solution *S* of (12) is bounded. More precisely, it satisfies:

$$\begin{aligned} 0 \le S(t,a) \le M f_\infty (a) < + \infty \quad \hbox {for all} \quad (t,a) \in {\mathbb {R}}^+ \times {\mathbb {R}}^+. \end{aligned}$$

### Proof

This result is a consequence of the maximum principle applied to *v* where $v(t,a) = S(t,a) / f_\infty (a)$. The evolution equation on *v* writes as

50 $$\begin{aligned} \begin{aligned}&f_\infty (a) \frac{\partial v(t,a)}{\partial t} + \beta (a) \frac{I(t)}{N} f_\infty (a) v(t,a) \\&\qquad \qquad \qquad = \frac{\partial }{\partial a} \left( D(a) f_\infty (a) \frac{\partial v(t,a)}{\partial a} \right) \ \ \hbox {for} \ \ (t,a) \in {\mathbb {R}}^+\times {\mathbb {R}}^+ ,\\&\frac{\partial v(t,0)}{\partial a} = 0 \ \ \hbox {for} \ \ t \in {\mathbb {R}}^+. \end{aligned} \end{aligned}$$

Hence,

51 $$\begin{aligned} \begin{aligned}&\frac{\partial v(t,a)}{\partial t} + \beta (a) \frac{I(t)}{N} v(t,a) - \frac{\partial }{\partial a} \left( D(a) \frac{\partial v(t,a)}{\partial a} \right) \\&\qquad \qquad \qquad = D(a) \frac{f_\infty '(a)}{f_\infty (a)} \frac{ \partial v(t,a)}{\partial a} \ \ \hbox {for} \ \ (t,a) \in {\mathbb {R}}^+\times {\mathbb {R}}^+ ,\\&\frac{\partial v(t,0)}{\partial a} = 0 \ \ \hbox {for} \ \ t \in {\mathbb {R}}^+. \end{aligned} \end{aligned}$$

Then, by the parabolic maximum principle in ${\mathbb {R}}^+$, since $v(0,a)\le M$ it follows that $v(t,a)\le M$ on ${\mathbb {R}}^+$ for all $t>0$. Indeed, we use the fact that for some positive constant *C*

$$\begin{aligned} \left| \frac{f_\infty '(a)}{f_\infty (a)}\right| \le C(1+a) \quad \hbox {and} \quad \beta (a) \le C(1+a^2). \end{aligned}$$

$$\square $$

Let us observe that it means that the probability distribution *f*(*t*, *a*) is bounded from above by a distribution proportional to $f_\infty$, i.e.

$$\begin{aligned} 0 \le f(t,a) \le \frac{M}{{\overline{S}}(t)} f_\infty (a) \ \ \hbox {for} \ \ (t,a) \in (0,T) \times {\mathbb {R}}^+, \end{aligned}$$

which leads to the justification of formal computations performed in Sect. 3 since ${\overline{S}}(t)$ decreases with *t* and remains positive.

Note also that we are able to obtain $H^1$ type regularity properties on *f* or more precisely on $v = S/f_\infty$

### Theorem 8

Assuming (*H*2) and (*H*4), and that the initial profile of susceptibles $S_0$ is such that $v_0=S_0/f_\infty$ satisfies

$$\begin{aligned} v_0 \in L^2({\mathbb {R}}^+; \nu ) \ \ \hbox {where} \ \ d \nu (a) = f_\infty (a) \, da. \end{aligned}$$

Then, the solutions *v* of Eq. (50) satisfy $v \in L^\infty ({\mathbb {R}}^+; L^2({\mathbb {R}}^+;\nu ))$, $\partial _a v \in L^2({\mathbb {R}}^+; L^2({\mathbb {R}}^+;\nu ))$ and

$$\begin{aligned} \frac{1}{2} \sup _{t \ge 0} \left\| v(t, \cdot ) \right\| ^2_{L^2({\mathbb {R}}^+;\nu )} + D_* \int _0^{+\infty } ds \left\| \frac{\partial v(s, \cdot )}{\partial a} \right\| ^2_{L^2({\mathbb {R}}^+;\nu )} \le \frac{1}{2} \left\| v_0 \right\| ^2_{L^2({\mathbb {R}}^+;\nu )}. \end{aligned}$$

### Proof

This result is the consequence of multiplication of Eq. (50) by *v* and integration by parts, using the fact that *v*, $\beta$ and *I* are non-negative. $\square$

We leave open the derivation of such estimates on the behavior at infinity in the more general case of System (12).

## Footnotes

1 It is often denoted ${{\mathcal {E}}}(f | f_\infty )$.

2 The corresponding python code and parameters are available at https://github.com/bdsbds/20220819_JMB/.
